# Genome-wide identification and analysis of anthocyanin synthesis-related R2R3-MYB genes in *Cymbidium goeringii*

**DOI:** 10.3389/fpls.2022.1002043

**Published:** 2022-09-28

**Authors:** Jiating Chen, Yuan-Yang Bi, Qian-Qian Wang, Ding-Kun Liu, Diyang Zhang, Xiangqing Ding, Zhong-Jian Liu, Shi-Pin Chen

**Affiliations:** ^1^College of Landscape Architecture, Fujian Agriculture and Forestry University, Fuzhou, China; ^2^Key Laboratory of National Forestry and Grassland Administration for Orchid Conservation and Utilization at College of Landscape Architecture, Fujian Agriculture and Forestry University, Fuzhou, China; ^3^College of Forestry, Fujian Agriculture and Forestry University, Fuzhou, China

**Keywords:** *Cymbidium goeringii*, orchids, gene family, R2R3-MYB, flower color, anthocyanin synthesis

## Abstract

The MYB gene family plays a vital regulatory role in plant metabolism, stress response, and floral color. The R2R3-MYB gene family of *C. goeringii* was identified, and its expression was analyzed using bioinformatics in this article. The R2R3-MYB genes of *Arabidopsis thaliana* were used as a reference to determine 104 *CgMYB* genes and categorize them into 22 subfamilies. Exon/intron organizations and conserved motif analysis revealed that the majority of *CgMYB* genes were highly conserved, and chromosome localization and collinearity analysis provided evidence of tandem duplication and segmental duplication events, indicating the phenomenon of gene family expansion and contraction. The function of *CgMYB* genes was analyzed by *cis*-acting element and gene ontology (GO) enrichment. In addition, we selected *CgMYB91* and *CgMYB32* for RT–qPCR, suggesting that *CgMYB91* and *CgMYB32* are associated with anthocyanin formation. In short, this study provides a comprehensive and specific function of the R2R3-MYB transcription factors (TFs) in orchids.

## Introduction

Transcription factors (TFs) are mostly engaged in regulating plant growth and metabolism. The avian myeloblastosis viral oncogene homolog (*v-MYB*, MYB) was first identified in vertebrates and widely found in eukaryotes (Smita et al., 2015; Zhao et al., 2020a). In plants, the MYB gene was first identified in maize (*Zea mays*) and named *ZmMYBC1* in Paz-Ares et al. (1987). Subsequently, it was identified and studied in model species such as *A. thaliana* and *Oryza sativa*, and the functions of multiple MYB genes were identified. The MYB TF family influences many aspects of plant growth processes. It plays a critical role in regulating various environmental stress responses and secondary metabolism, also controlling the morphology and pattern of the cells (Lloyd et al., 2017; Li J. et al., 2019; Li X. et al., 2019; Cao et al., 2020; Chen H. et al., 2020). As a superfamily, MYB TFs can be categorized into four subgroups based on their one to four conserved MYB domains (R domains), called 1R-MYB factors (MYB-related), R2R3-MYB factors, 3R-MYB factors, and 4R-MYB factors. The R domain consists of approximately 52 amino acids that form three alpha-helices, containing one tryptophan for every 18 amino acids. The common feature of MYB TFs is that they all have DNA binding domains at the N-terminus of the protein.

One of the most prominent groups of MYB TFs is the R2R3-MYB family in plants (Hou et al., 2018a). R2R3-MYB involves conserved DNA-binding domains with five conserved tryptophan residues (Fan et al., 2020). The R2R3-MYB protein is highly variable in regions other than the conserved MYB structural domain, so it has many functions. R2R3-MYB TFs (126 members) were divided into 25 subfamilies (S1-S25) in *A. thaliana*, mainly based on the different regulatory roles of TFs, and their functions remain conserved in other species (Matus et al., 2008; Dubos et al., 2010). R2R3-MYB can regulate multiple functions during plant growth. For example, in all of these subgroups, the S6 subfamily is associated with anthocyanin synthesis (Plunkett et al., 2018; Yan et al., 2021). *AtMYB21* (S19) and its homologs regulate flavonol synthesis by regulating *FLS1* gene expression (Zhang et al., 2021). *AtMYB44* (S22) negatively controls the performance of the ABA-responsive gene *RAB18* (Li et al., 2014). *AtMYB66* (S15) controls root hair initiation with BHLH (basic helix-loop-helix) TFs and WD40 (WD-repeat) proteins (Grierson et al., 2014). *AtMYB96* (S1) increases plant drought tolerance by controlling cuticular wax biosynthesis under drought conditions (Chen et al., 2018). *MYB117* from poplar promotes anthocyanin synthesis by upregulating the flavonoid 3′,5′-hydroxylase gene (Ma et al., 2021). Several R2R3-MYB genes also governed cotton fiber initiation (MacHado et al., 2009). In conclusion, some MYB TFs of the R2R3 type regulate plant-specific activities.

Pigments in plants mainly include flavonoids, carotenoids, and chlorophyll. Anthocyanins are the main flavonoids involved in flower color formation, which also play a role in stress and defense (Zaidi et al., 2019). Anthocyanins, water-soluble pigments, are catalyzed through a variety of enzymes in the biosynthesis pathway, including phenylpropanoid biosynthesis, the early flavonoid pathway, and the specific anthocyanin pathway (Shi and Xie, 2014). In the metabolic pathway of anthocyanins, some TFs influence the performance of structural genes and control the formation of anthocyanins. R2R3-MYB protein regulates anthocyanin biosynthesis with bHLH and WD40 proteins, which generally form MYB-bHLH-WD40 complexes (Allan et al., 2008; Montefiori et al., 2015; Yan et al., 2021). The *Pr* genes in purple cauliflower (Brassica oleracea var. botrytis) promote the accumulation of anthocyanins, causing the formation of purple pigments (Chiu et al., 2010). *MYBA* and BHLH genes are responsible for anthocyanin expression in blueberry (*Vaccinium* section *Cyanococcus*) (Plunkett et al., 2018). *OgMYB1* can regulate the expression of anthocyanin biosynthetic genes to form red spots in yellow lip tissue in *Oncidium* Gower Ramsey (Chiou and Yeh, 2008). Transient overexpression of two R2R3-MYB (*RcPAP1* and *RcPAP2*) of *Rhyncholaeliocattleya* Beauty Girl “KOVA” could activate ABP structural genes and promote red pigmentation in Phalaenopsis flowers (Li et al., 2020). *PyMYB10* has been proven to be related to pigment synthesis in pear skins (Feng et al., 2010). At the same time, several R2R3-MYBs have been successively shown to be involved in anthocyanin biosynthesis in *Phalaenopsis* spp. (Hsu et al., 2015), *Eutrema salsugineum* (Qi et al., 2020), *Prunus mume* (Zhang Q. et al., 2017), *Sapium sebiferum* (Chen et al., 2021a), and *Myrica rubra* (Shi et al., 2021).

*Cymbidium goeringii* is a terrestrial orchid species of Orchidaceae and is a typical *Cymbidium* species in China. Orchids are highly ornamental with their specialized lip to attract pollinators, so their lip usually has brightly colored spots or unique shapes. *C. goeringii’s* flower colors are simple, but the unique purple–red lip spots are also attractive. A previous study found that flower color metabolic pathways include carotenoid and anthocyanin in *C. goeringii* (Sun et al., 2021). In this study, we identified the R2R3-MYB gene family in *C. goeringii* and analyzed their classification, phylogenetics, and expression model. We also explored some genes involved in flower colors. Our findings will provide valuable information for further studies of *C. goeringii* and other orchids.

## Materials and methods

### Plant materials

The wild *C. goeringii* acquired in this study were cultivated at “Forest Orchid Garden” in Fujian Agriculture and Forestry University (Fuzhou, Fujian Province, China) under the shade of trees with natural light and temperatures. This study used the floral organs (sepals, petals, lips, columns) of pale-yellow flowers with purple-red spots (YF) and light pale-red flowers (RY) during the bloom period. All samples were collected and frozen in tubes, placed at −80°C, and set aside.

### Identification of R2R3-MYB genes family

The R2R3-MYB proteins of *A. thaliana* was downloaded from the TAIR database.^1^ A BLAST method was used to identify potential R2R3-MYB proteins (Chen Y. et al., 2020). It used a BLAST algorithm (*E*-value < 1e-3) and AtMYB proteins as a reference to search the R2R3-MYB members by using TBtools (Zhang C. et al., 2017; Chen C. et al., 2020). Then, all possible sequences were once again BLASTP on NCBI^2^ (Zhang C. et al., 2017; Wang et al., 2021). The number Pfam (PF00249) represents the MYB-like DNA-binding domain, so the hidden Markov model (HMM) was used to generate genes with conserved MYB domains by a simple HMM search program in TBtools software (Stracke et al., 2001). In addition, candidate gene sequences that were identified previously were further confirmed in the Pfam,^3^ NCBI-CDD,^4^ and SMART^5^ databases. The primary structure of proteins was analyzed on ExPASy (Expert Protein Analysis System),^6^ including molecular weight (Mw), theoretical isoelectric point values (pI), grand average of hydrophilic (GRAVY), aliphatic index (AI), and instability index (II) (Artimo et al., 2012). The website Euk-mPLoc 2.0 (Euk-mPLoc 2.0 server)^7^ was used to predict the subcellular localization (Chou and Shen, 2010).

### Phylogenetic analysis of the *CgMYB* genes

The alignment sequences included 104 CgMYB proteins and 126 AtMYB proteins, three PeMYB proteins in *Phalaenopsis* spp., one OgMYB protein in *Oncidium* Gower Ramsey, two RcPAP proteins, one RcPCP protein in *Cattleya hybrid* “KOVA” and one OsMYB protein in *O. sativa*. The sequences of CgMYB proteins are listed in Supplementary Table 1. We downloaded 126 *AtMYB* sequences from the TAIR database. R2R3-MYB sequences of other plants were acquired from GenBank (see text footnote 2), and the gene accession numbers of the sequences are as follows: *PeMYB2*/*PeMYB11*/*PeMYB12* (KF769467/KF769476/KF769477), *OgMYB1* (EF570115), *RcPAP1/RcPAP2* (MN420461/MN420462), *RcPCP1* (MN420463), and *OsMYB112* (AK104457). The phylogenetic relationship among the aligned sequences obtained was estimated by the neighbour-joining (NJ) method by MEGA 5 software. In detail, the alignment sequences selected with the MUSCLE method, Gap Open and Gap Extend, are −2.9 and 0, respectively; other values keep the default. Then, the Phylogeny test was performed using 1,000 replications of the bootstrap method. The Poisson model and uniform rates are adopted in the substitution model and rates and patterns, respectively. Gaps/missing data treatment with pairwise deletion (Tamura et al., 2011; Fan et al., 2020). In previous studies, R2R3-MYB members of *A. thaliana* were divided into 25 subfamilies (Stracke et al., 2001; Dubos et al., 2010). The phylogenetic tree was exported in the Newick format file and visualized using EvolView^8^ (Subramanian et al., 2019).

### Conserved motif and gene structure analysis

Conserved motifs of R2R3-MYB proteins from *C. goeringii* were analyzed on the MEME website^9^ (Bailey et al., 2009). We analyzed ten motifs in this paper. The exon–intron structure of the *CgMYB* genes was analyzed *via* TBtools with Gene Location Visualize. The images were then combined with Adobe Illustrator software.

### R domain analysis of the CgMYB proteins

The R2 and R3 domains of 104 CgMYB proteins were aligned using MEGA 5. The conserved domain regions were submitted to the WebLogo website^10^ to acquire sequence logos (Crooks et al., 2004). The size of amino acids in the figure is related to the conservation degree of the sequence. The sequences of the S6 subfamily in this study were compared on the website European Bioinformatics Institute (EMBL-EBI)^11^ using the online tool Clustal Omega, and the results were edited with Jalview software.

### Chromosome localization analysis, collinearity analysis, and *Ka/Ks* analysis

Information on the distribution of *CgMYB* genes on the chromosomes was analyzed *via* TBtools software. Collinearity analysis among the *C. goeringii* chromosomes was performed using TBtools with the One Step MCScanx command. The segmental duplications among *CgMYB* genes were displayed with the Advance Circos package program in TBtools. Then, *Ka/Ks* analysis was calculated by TBtools software (Chen C. et al., 2020).

### Analysis of the promoter elements of the *CgMYB* genes

The upstream 2000 base pairs sequence of the *CgMYB* gene was extracted using TBtools software, and the *cis*-acting element was predicted in the PlantCARE database^12^ (Lescot et al., 2002).

Then, the data were processed using Excel software, followed by TBtools and Origin 2018 software for visualization.

### Different expression patterns and gene ontology classification analysis of the *CgMYB* genes

The gene expression levels of different tissues (petals, sepals, lips, and columns) in pale-yellow and light purple-red flowers were analyzed. The raw RNA-Seq data from *C. goeringii* were obtained from the National Center for Biotechnology Information (NCBI) database with BioProject accession number PRJNA749652. The FPKM values of *CgMYB* genes were visualized by TBtools. Then, Gene Ontology (GO) classification analysis of *CgMYB* genes based on transcriptome data was performed, and the image was generated using Origin 2018 software.

### The real-time reverse transcription quantitative polymerase chain reaction experiment on different flower tissues

Total RNA was extracted from different flower tissues using the Biospin Polysaccharide Polyphenol Plant Total RNA Extraction Kit (BioFlux, Beijing, China). Reverse RNA transcription was performed with TransScript^®^ All-in-One First-Strand cDNA Synthesis SuperMix for quantitative PCR (qPCR; TransGen Biotech, Beijing, China). Then, we used the TranScript^®^ All-in-One First-Strand cDNA Synthesis SuperMix for PCR to remove the genomic DNA. Two genes were selected for quantitative fluorescence verification, and their DNA sequences are listed in Supplementary Table 2. In previous studies, *PeMYB11* (S6 subfamily) controls the red spots on the labellum of *Phalaenopsis* and the expression of *OsMYB112* (S4 subfamily) is consistent with anthocyanin content (Hsu et al., 2015; Xiang, 2016). We speculated that *CgMYB91* (S6 subfamily) and *CgMYB32* (S4 subfamily) are associated with the synthesis of anthocyanins and selected them for the next RT-qPCR experiment. Corresponding primers for the two *CgMYB* genes were designed using Premier 5.0 software, and the primers are shown in Supplementary Table 3. Additionally, primer specificity was checked through the NCBI website with Primer-BLAST. RT–qPCR analysis was performed using 2X Universal SYBR Green Fast qPCR Mix (ABclonal, Wuhan, China). The Actin gene was used as an internal reference (Xia and Liu, 2020). Three biological replications of each sample were used for RT–qPCR analysis, and the expression of two genes in different flower tissues was calculated by the 2^–ΔΔCt^ method (Feng et al., 2011).

## Results

### Identification and physicochemical property analysis of the *CgMYB* genes

We discovered 104 R2R3-MYB genes in *C. goeringii* and called them each from *CgMYB1* to *CgMYB104*. The lengths of the *CgMYB* genes varied from 471 base pairs (*CgMYB72*) to 2160 base pairs (*CgMYB90*), while the average size of the CgMYB proteins was 292.45 amino acids, extending from 156 to 869. The Mw of CgMYB proteins ranged from 17.64 kDa for *CgMYB72* to 99.48 kDa for *CgMYB90*. The theoretical isoelectric point (pI) values varied from 4.76 (*CgMYB57*) to 10.87 (*CgMYB38*), while the anticipated GRAVY values ranged from −1.022 (*CgMYB31*) to −0.277 (*CgMYB90*), indicating that most CgMYB proteins are hydrophilic. Furthermore, the AI of *CgMYB*-derived proteins varied from 56.68 for *CgMYB97* to 97.15 for *CgMYB11*. The II ranged from 41.34 for *CgMYB91* to 79.35 for *CgMYB104*, all over 40, indicating that most CgMYB proteins are instability proteins. The predicted subcellular localization revealed that 90 (86.53%) of the 104 CgMYB proteins may be located in the nucleus, 13 in the nucleus and cytoplasm, and one in the cytoplasm. The specific genes and corresponding protein information can be found in Supplementary Table 4.

### Phylogenetic analysis of the *CgMYB* proteins

**FIGURE 1:**
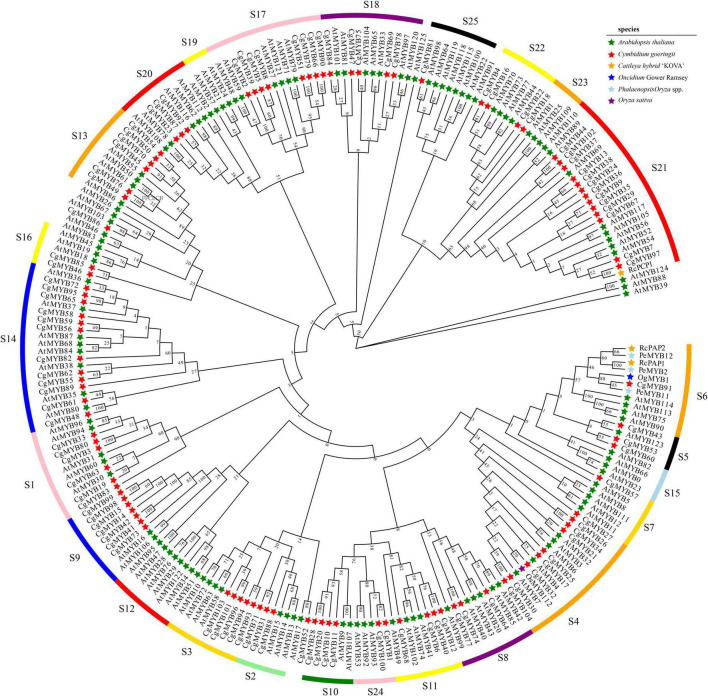
Phylogenetic analysis of the *CgMYB* genes. In total, 104 *CgMYBs* from *Cymbidium goeringii* and 126 *AtMYBs* from *Arabidopsis thaliana*, as well as *PeMYB2*, PeMYB11, and PeMYB12 in *Phalaenopsis* spp. and *OgMYB1* in *Oncidium Gower Ramsey* as well as RcPAP1, RcPAP2, and RcPCP1 in the *Cattleya hybrid* “*KOVA”* and *OsMYB112* in *Oryza sativa* were used to generate the NJ tree. The subfamilies of S1–S25 represented subfamilies based on groups of R2R3-MYB proteins in *A. thaliana*.

Multiple alignments of the amino acid sequences from six species, *C. goeringii*, *A. thaliana*, *Phalaenopsis* spp., *Oncidium* Gower Ramsey, *Cattleya hybrid* “KOVA,” and *O. sativa*, were used (Chiou and Yeh, 2008; Hsu et al., 2015; Xiang, 2016; Li et al., 2020). According to Figure 1, phylogenetic analysis showed that 104 CgMYBs were divided into 22 subfamilies with members ranging from 1 to 13, as previously studied (Hou et al., 2018b). Among the 22 subfamilies, the S21 subfamily includes the most members (13), followed by the S14 subfamily (12) and the S4 subfamily (nine). Subfamilies S17 and S9 comprised eight and seven members, respectively. Six individuals made up two subfamilies (S1 and S13). The two subfamilies are all five members, including S3 and S18. Subfamilies S11 and S10 have four members, and subfamilies S16 and S24 have two members. Subfamilies S2, S5, S8, S20, S22, S6, S7, S23, and S25 all contained three members or one member, respectively. However, no genes were categorized into subgroups S12, S15, and S19, suggesting that the majority of R2R3-MYB genes are distributed similarly to other orchids (Fan et al., 2020).

### Conserved motif and gene structure analysis

**FIGURE 2:**
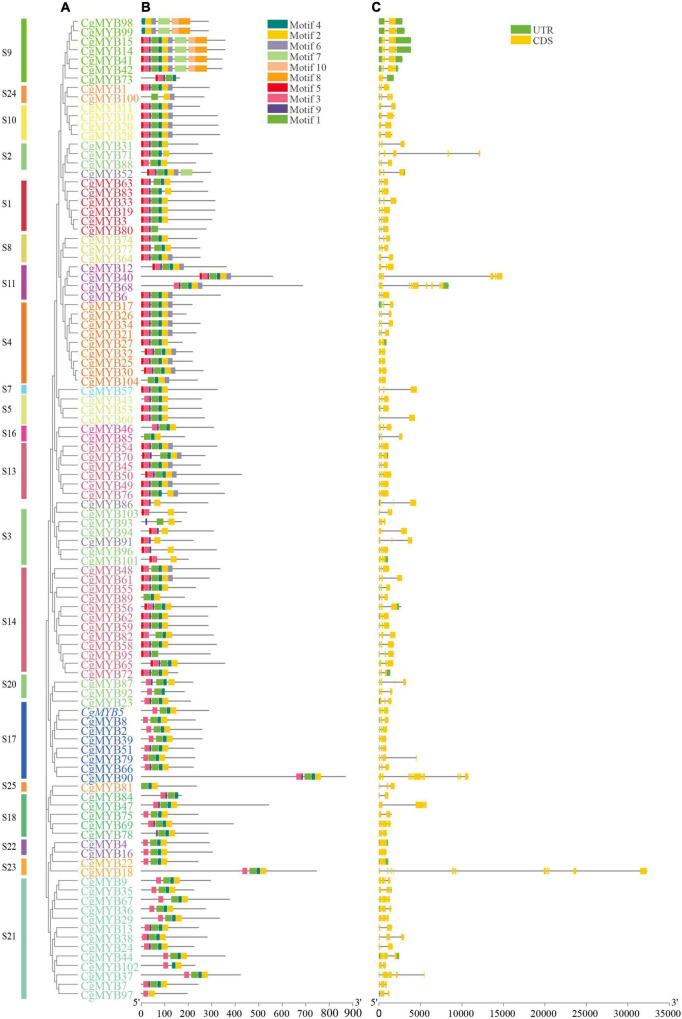
Conserved motif and gene structure of the *CgMYB* genes. **(A)** Phylogenetic tree of 104 *CgMYB* proteins. **(B)** Ten conserved motifs in the *CgMYB* genes and represented by different colored squares. **(C)** UTR, exon, and intron structures of *CgMYB* genes in *Cymbidium goeringii*. The UTR (s), exon (s), and intron (s) are shown by the green square, yellow square, and gray line, respectively.

The NJ tree, gene structure analysis, and conserved motif structures of 104 *CgMYB* genes were displayed by TBtools (Figure 2). The results showed that the same branch or same subfamily had the same motifs and gene structures. A few genes have upstream and downstream UTR structures. Among them, 11 genes had both 5′UTR and 3′UTR, 17 had only 5′UTR, nine had only 3′UTR, and others did not have either structure. The number of exons in the R2R3-MYB gene of *C. goeringii* ranged from 1 to 12 and was mainly characterized by two to three exons. Sixty-six (63%) CgMYBs had three exons, 20 (19%) had two exons, eight genes had four exons, and only six genes had more than four exons. Introns separate most *CgMYB* (97%) sequences, and only three genes (*CgMYB4*, *CgMYB16*, *CgMYB22*) do not contain introns. More than half (59%) of the genes were spliced by two introns and three exons, while 18 *CgMYB* genes had one intron and two exons, with the remaining genes ranging from 3 to 12. Figure 2 shows that most of the sequences are within 10 kb in length, and a few are longer than 10 kb, mainly due to long introns, which are also found in other orchids (Ke et al., 2021). Although the measurements of introns and exons were not uniform, the distribution of their numbers implied that the sequence structures were highly similar in the same subfamily. For example, the S1 subfamily had a pattern of two introns and three exons, while the S4 subfamily had two introns and three exons or one intron and two exons. Ten conserved motifs were identified by the MEME suite server, and the information on motifs 1–10 are shown in Supplementary Table 5. Among them, motif 2 forms a complete R3 domain, and motifs 1, 3, and 9 form a complete R2 domain. Most *CgMYBs* have motifs 1, 2, 3, 4, 5, and 9, while some families have their own unique motif structure. For example, the S11 and S4 subfamilies, located in the same branch, have motifs 7 except *CgMYB68*, while the S9 subfamily has their characteristic motifs 8 and 10. This result implies that it may have a different regulatory function than other genes.

### R domain analysis of the *CgMYB* proteins

**FIGURE 3:**
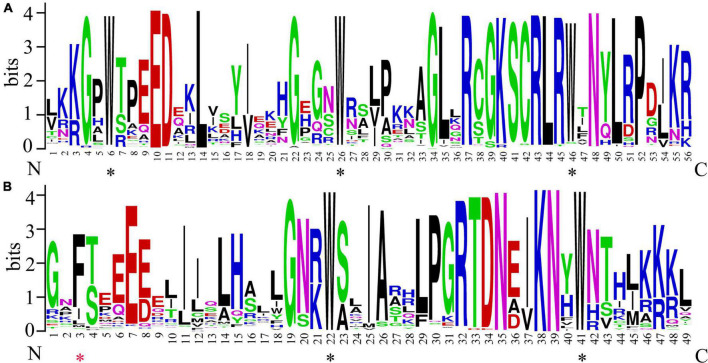
Conserved domain sequence of the *CgMYB* genes. **(A)** The sequence logos of the R2 conserved domain include three conserved tryptophan residues. **(B)** Sequence logos of the R3 conserved domain; the first tryptophan (W) residues are always replaced by phenylalanine (F) Black * represents typically conserved tryptophan amino acid sites, and red * represents the substituted amino acid site in the R3 domain.

To analyze the conserved position in *CgMYB* sequences, the sequence logo was generated by the WebLogo network. Picture A represents the R2 respect, and picture B represents the R3 respect (Figure 3). We found that the conserved tryptophan (Trp, w) residues are approximately separated by 18 amino acids, as in a previous study. The R2 domain has three Trp residues, and only the second and third Trp residues were conserved in the R3 domain. The first Trp was always substituted by phenylalanine (F).

**FIGURE 4:**
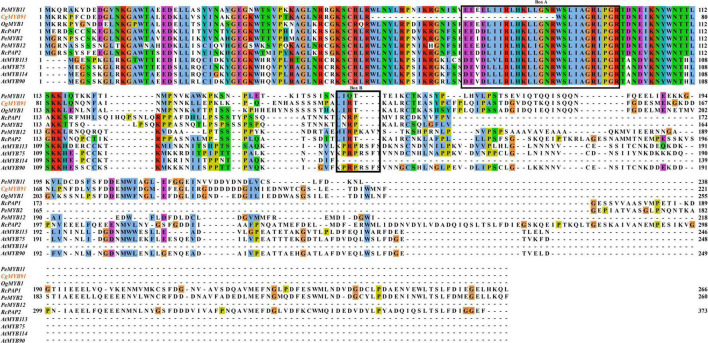
Results of multiple sequence alignment of the S6 subfamily. Sequences of *CgMYB91* from *Cymbidium goeringii* and four *AtMYBs* from *Arabidopsis thaliana*, as well as *PeMYB2*, *PeMYB11*, and *PeMYB12* in *Phalaenopsis* spp. and *OgMYB1* in *Oncidium* Gower Ramsey as well as *RcPAP1*, *RcPAP2*, and *RcPCP1* in the *Cattleya hybrid* “KOVA,” were aligned. Box **(A)** represents the bHLH-binding motif, and box **(B)** represents the conserved regions specific to anthocyanin regulation.

Figure 4 shows this study’s sequence alignment of the S6 subfamily. The bHLH-binding motif ([DE]Lx2[RK]x3Lx6Lx3R) of the *CgMYB91* sequence was partially missing. The anthocyanin-specific motif was present with the same amino acid arrangement (L-I-R-T) as the sequences of Cattleya and *Oncidium*, presumably regulating the formation of anthocyanins in *C. goeringii* (Stracke et al., 2001; Zimmermann et al., 2004; Chiou and Yeh, 2008; Hsu et al., 2015; Li et al., 2020).

### Chromosome localization analysis, collinearity analysis, and *Ka/Ks* analysis

**FIGURE 5:**
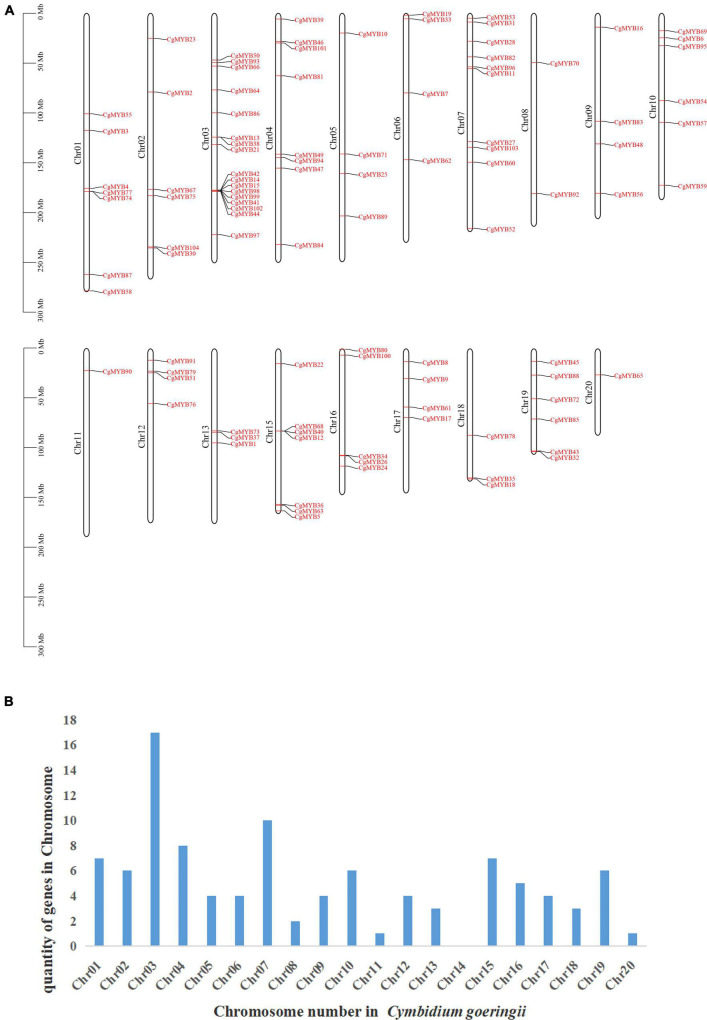
Results of chromosome localization. **(A)** The figure shows the distribution of *CgMYB* genes from *Cymbidium goeringii* on 20 chromosomes. **(B)** The figure shows the number of R2R3-MYB genes on chromosomes.

To further analyze the distribution of R2R3-MYB on chromosomes in *C. goeringii*, the GFF annotation file was used to analyze its chromosome information. According to Figure 5, *CgMYB* genes were found on all chromosomes except chromosome 14. A total of 102 *CgMYB* genes were located on the chromosomes, but two genes (*CgMYB20* and *CgMYB29*) were on the scaffolds and have not been mapped. Chromosome 3 has the largest number of R2R3-MYB genes, with 17 genes. This was followed by chromosome 7 with ten genes and chromosome 4 with eight genes. Chromosomes 1 and 15 have seven genes; chromosomes 2, 10, and 19 all have six genes; chromosome 16 has five genes; chromosomes 5, 6, 9, 12, and 17 also all have four genes; and chromosome 13 has three genes. In contrast, chromosome 8 has two genes. In addition, chromosomes 11 and 20 only have one gene.

Analysis of tandem repeat genes revealed the presence of tandem repeat genes on chromosome 1, chromosome 3, and chromosome 15. *CgMYB77* and *CgMYB74*, *CgMYB13* and *CgMYB38*, *CgMYB42* and *CgMYB41* and *CgMYB99* and *CgMYB98* and *CgMYB15*, *CgMYB102* and *CgMYB44*, and *CgMYB68* and *CgMYB40* and *CgMYB12* were located at the same point on the chromosome. They share the same conserved region and structure.

**FIGURE 6:**
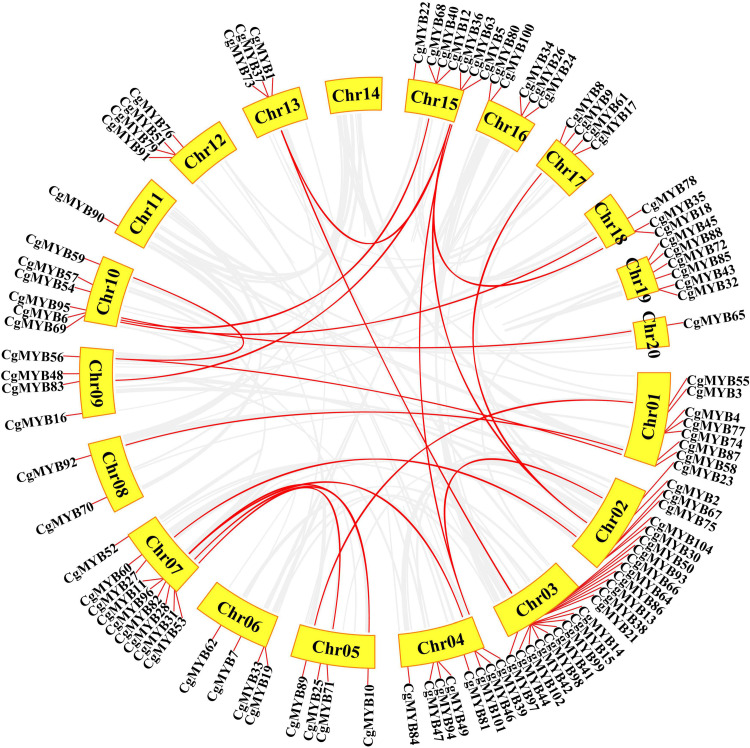
Synteny analysis of the *CgMYB* genes. Red lines represent the duplicated *CgMYB* gene pairs in the genome. The chromosome number is presented next to each chromosome.

According to Figure 6, the collinearity analysis showed 21 pairs of segmental duplications, with the most significant number of segmental duplications on chromosome 15, followed by chromosome 7. Chromosomes 3, 8, 17, and 20 had only one segmental duplication.

The *Ka/Ks* ratio of 22 collinear gene pairs (21%) was calculated (Supplementary Table 6). The *Ka/Ks* ratios of the gene pairs except for *CgMYB2*/*CgMYB39* and *CgMYB69*/*CgMYB78* were less than 1, which indicated that most R2R3-MYB genes mainly underwent negative selection pressure.

### Analysis of the promoter elements of the *CgMYB* genes

**FIGURE 7:**
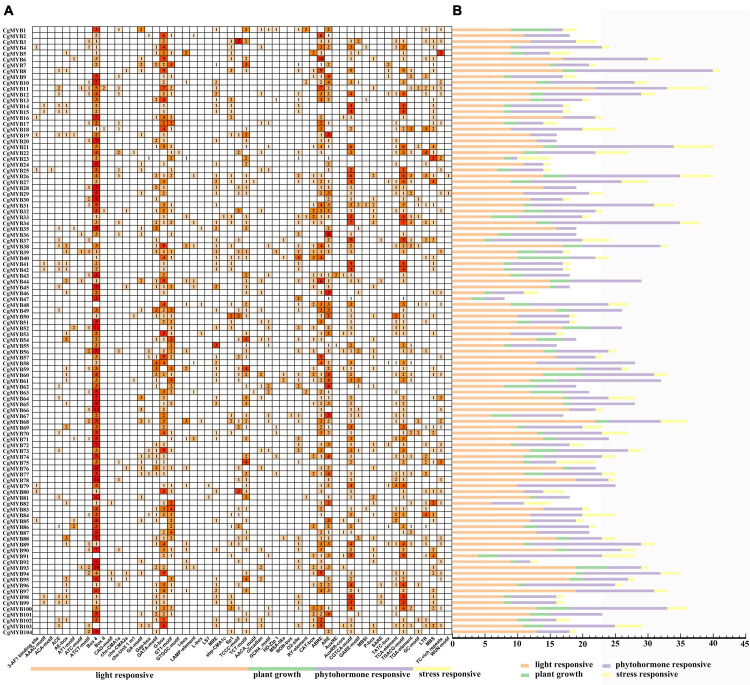
The result of promoter elements of the *CgMYB* genes. **(A)** Number of promoter elements in *Cymbidium goeringii*. **(B)** Orange, green, purple, yellow, bars represent the light-responsive, plant growth, phytohormone responsive and stress-responsive elements in *CgMYB* promoter regions, respectively.

The promoter elements were predicted in the 2000 base pairs upstream promoter regions of 104 *CgMYB* genes. According to Figure 7, the number of response elements of light, phytohormone, plant growth, and stress was 29, 13, nine, and five, respectively. Among them, the maximum number of light-response elements, especially Box 4 (399/1245, 32%) and G-box elements (214/1245, 17%), was followed by 98 GT1 motifs, 96 TCT motifs, 67 GATA motifs, etc. Among the plant growth- and development-related response elements, CAT-box (50/173, 29%) and O2-site (54/173, 31%) were associated with meristematic tissue expression and arginine metabolism, respectively. In contrast, other elements, such as the AACA motif, circadian rhythm, RY element, and GCN4 motif, were associated with seed regulation, endosperm expression, and circadian control. Among the phytohormone response elements, ABRE (183/863, 21%), ARE (158/863, 18%), CGTCA-motif (175/863, 20%), and TGACG-motif (175/863, 20%) were more numerous and were associated with abscisic acid response, anaerobic induction, and MeJA, respectively, while the remaining elements were associated with salicylate response, flavonoid synthesis and auxin response. The stress response components included five features, MBS, GC-motif, LTR, TC-rich repeats, and WUN-motif, which are related to drought, hypoxia, cold, wound stress, etc. Therefore, the R2R3-MYB genes are mainly associated with plant growth, stress, and tissue metabolism regulation.

### Different expression patterns and gene ontology classification analysis of the *CgMYB* genes

**FIGURE 8:**
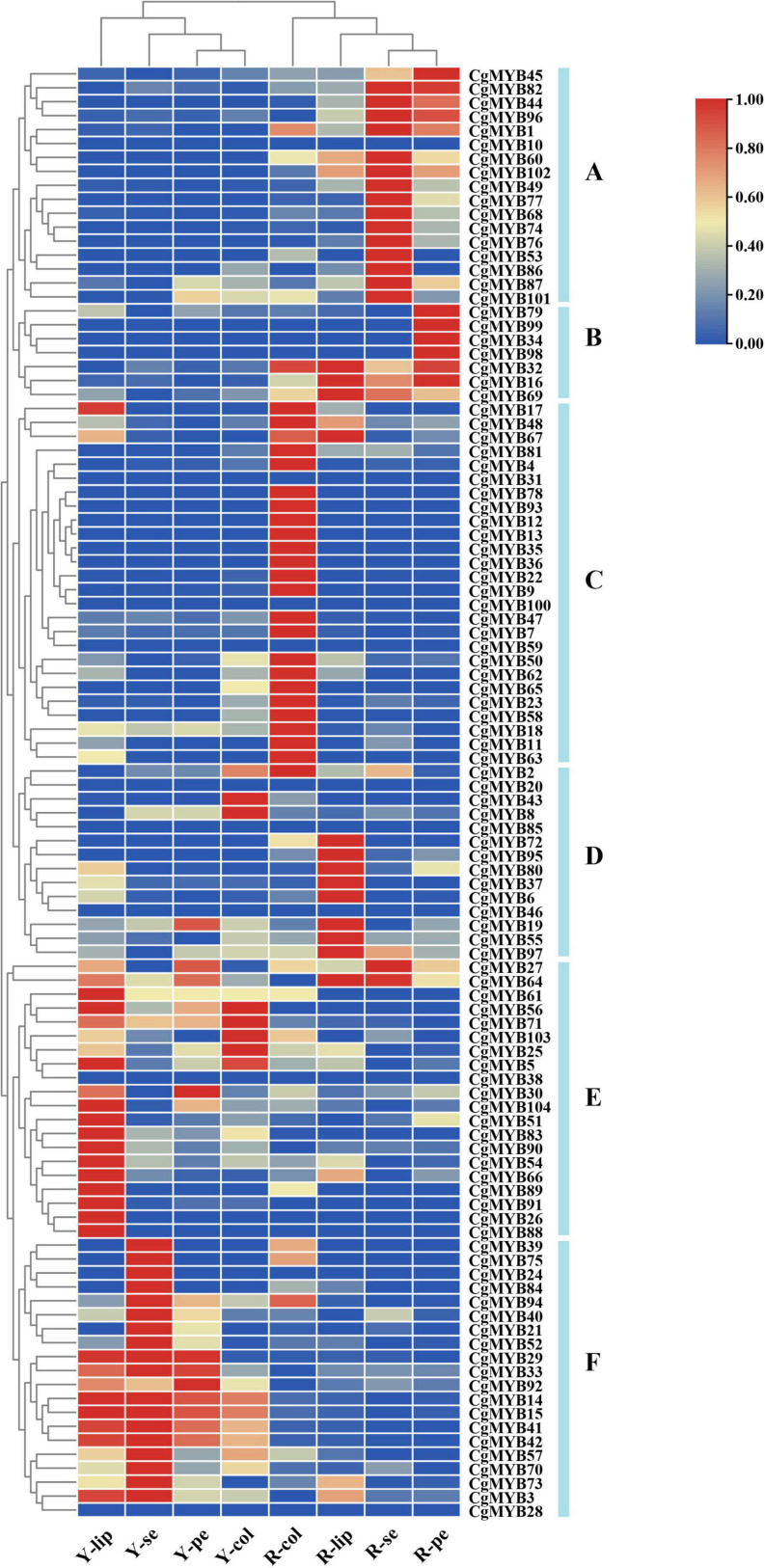
Expression of *CgMYB* genes on different flower tissues. A total of 104 *CgMYB* genes were classified into six groups **(A–F)** based on their expression levels in different flower tissues. The expression level increased gradually from blue to red. The heatmap was plotted from the FPKM value and performed with min-max normalization. Red indicates high levels of expression, while blue indicates low levels of expression. Y-se, sepals of pale-yellow flowers with purple-red spots; Y-pe, petals of pale-yellow flowers with purple-red spots; Y-lip, lips of pale-yellow flowers with purple-red spots; Y-col, columns of pale-yellow flowers with purple-red spots; R-se, sepals of the light purple-red flower; R-pe, petals of the light purple-red flower; R-lip, lips of the light purple-red flower; R-col, columns of the light purple-red flower.

Based on the transcriptome data, the expression profile of 104 *CgMYB* genes in different tissues, such as sepals, petals, lips, and columns of pale-yellow flowers with purple-red spots (YF) and light purple-red flowers (RF). And their FKFM values are listed in Supplementary Table 7. For the expression heatmap, 104 genes were classified into six groups (A-F) (Figure 8). A total of 17 genes are located in Group A and are mainly expressed in the sepals and petals in RF. Group B contains seven genes expressed primarily in RF, especially in the petals. Group C includes 26 genes, primarily expressed in the RF column. Fourteen genes were in Group D, expressed primarily at the lip in RF, while 20 genes in Group E were mainly expressed in the lips, petals, and columns in YF. Group F has 20 genes commonly expressed in the lips, sepals, petals, and columns in YF.

**FIGURE 9:**
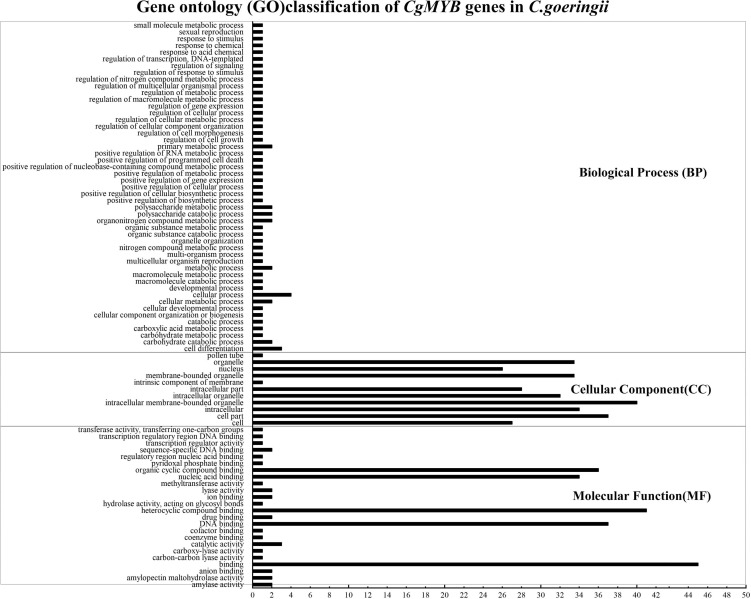
Gene ontology analysis of *CgMYB* genes. The figure shows the enrichment of *CgMYB* genes in different functions. The annotation information is listed in Supplementary Table 8.

Gene ontology annotation was mainly divided into biological processes, cellular components, and molecular functions (shown in Figure 9). The annotation information is listed in Supplementary Table 8. The majority of genes were enriched in cellular components and biological processes. In cellular components, genes were enriched in intracellular membrane-bound organelles, membrane-bound organelles, bound organelles, cell parts, organelles, etc. At the same time, in biological processes, most of the genes were mainly enriched in nucleic acid binding, organic cyclic compound binding, binding, DNA binding, etc.

### The real-time reverse transcription quantitative polymerase chain reaction experiment on different flower tissues

**FIGURE 10:**
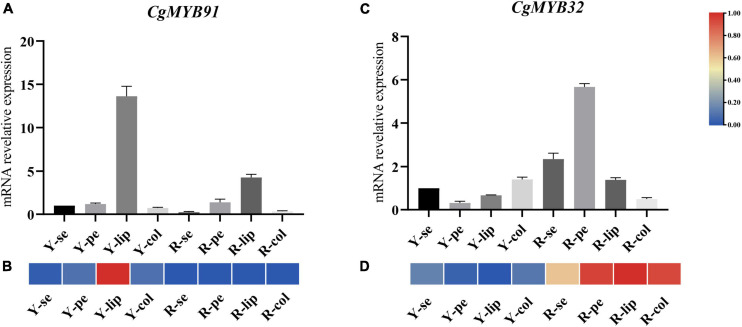
Real-time reverse transcription quantitative polymerase chain reaction (RT–qPCR) on different flower tissues. **(A,B)** The relative expression levels at different floral tissues and corresponding expression pattern of *CgMYB91*; **(C,D)** the relative expression levels at different floral tissues and corresponding expression pattern of *CgMYB32*. The error bars represent the standard deviations of three biological replicates. The expression pattern decreased gradually from red color to blue color. Y-se, sepals of pale-yellow flowers with purple-red spots; Y-pe, petals of pale-yellow flowers with purple-red spots; Y-lip, lips of pale-yellow flowers with purple-red spots; Y-col, columns of pale-yellow flowers with purple-red spots; R-se, sepals of the light purple-red flower; R-pe, petals of the light purple-red flower; R-lip, lips of the light purple-red flower; R-col, columns of the light purple-red flower.

Samples were taken at several flower organs in two flower color types of *C. goeringii*: sepals, petals, lips, and columns of pale-yellow flowers with purple-red spots (YF) and light purple-red flowers (RF). Previous studies suggested that the S6 subfamily was closely related to anthocyanin formation, and some R2R3-MYB TFs related to anthocyanin synthesis were also identified in S4 subfamilies. *CgMYB91* (S6) and *CgMYB32* (S4) were selected to further explore the expression of genes related to anthocyanin synthesis in the R2R3-MYB family. The experimental data is shown in Supplementary Table 9. The expression of *CgMYB91* was relatively high on the lips, especially in the pale-yellow flowers with purple-red spots (YF), and low in the other parts of the flowers. The expression of *CgMYB32* was significantly in the RF’s sepals, petals, and lips, particularly in the petals (shown in Figure 10). RT-qPCR expression levels of both genes were generally consistent with transcriptome expression.

## Discussion

*Cymbidium goeringii* is a critical ornamental species in the orchid family with various flower colors, floral scents and flower types. Flower color has an important value in plant pollination and the application of ornamentals (Renoult et al., 2017). The metabolic pathway of flower color formation has been studied in many plants. R2R3-MYB TFs have a vital function in anthocyanin synthesis, mainly through regulating the expression of the ABP structural gene (Lloyd et al., 2017).

This study identified 104 *CgMYBs* from *C. goeringii* of R2R3-MYB genes and divided them into 22 subfamilies. Compared to other orchids, the number of R2R3-MYB genes identified was similar to that in other orchids, such as *Cymbidium ensifolium* (102), D*endrobium officinale* (101), *Phalaenopsis aphrodite* (99), and *Phalaenopsis equestris* (96) (Wang et al., 2018; Fan et al., 2020; Ke et al., 2021). However, the number of R2R3-MYB genes identified was less than those in *A. thaliana* (126), potato (112), moso bamboo (114), grapes (108), and poplar (207), which may be related to replication events in the genome and reflect differences between orchids and other plants (Stracke et al., 2001; Matus et al., 2008; Hou et al., 2018b; Li X. et al., 2019; Zhao et al., 2020b).

The 104 *CgMYBs* were classified into 22 subfamilies in *C. goeringii* due to similar conserved regions. Different subfamilies have corresponding regulatory functions in the R2R3-MYB gene family. *CgMYB91* was clustered with *AtMYB75*, *AtMYB90*, *PeMYB11*, *PeMYB12*, etc., in the S6 subfamily, speculating that it regulates anthocyanin synthesis. *CgMYB32* and *AtMYB4*, and *OsMYB112* in S4, which suggests that *CgMYB32* is related to anthocyanin formation and may be an inhibitor of the ABP pathway. *CgMYB43* was clustered with *AtMYB123* (subgroup 5) and may be related to root development (Liu et al., 2012). Thus, *CgMYB91* and *CgMYB32* will be further studied. At the same time, clustering results of the R2R3-MYB gene from *C. goeringii* and *A. thaliana* as in *C. ensifolium*/*D. officinale*/*P. aphrodites*, most genes were found in the S21 subfamily, and no members were found in the S15 and S12 subfamilies, implying that no MYB TFs are involved in root hair regulation and glucosinolate biosynthesis in Orchidaceae (Gigolashvili et al., 2009; Grierson et al., 2014). In addition, some genes were not included in the 25 subgroups, suggesting that they may have different functions from *A. thaliana*. The reason may relate to the duplication and loss of genes during the evolution of orchids, which also predicts specificity in these four orchids.

Most of the *CgMYBs* contain motifs 1, 2, 3, and 9. The composition and distribution order of motifs are similar in branches or subfamilies of the phylogenetic tree, and the composition of motifs varies significantly in different groups. Only the S9 subfamily has motifs 7, 8, and 10, suggesting that it has different functions from the other subfamilies. This result has also been found in *C. ensifolium* and other plants (Ke et al., 2021). The majority of R2R3-MYB (∼59%) contains two introns and three exons, and the same is found in other plants (Hou et al., 2018b; Fan et al., 2020; Chen et al., 2021b; Ke et al., 2021). Three *CgMYB* genes have more than six introns, and the multiple non-coding regions may give them complex structures and functions, but most of the sequences are conserved in introns and exons. R2R3-MYB contains two conserved domains, the R2 and R3 domains. The first tryptophan of the R3 structural domain is mostly replaced by phenylalanine (F), which is also the case in other plants.

A combination of genome replication, segmental duplications, and tandem duplications is considered the major evolutionary force (Magwanga et al., 2018). The evolution of poorly adapted plants causes a rise or decline in the number of gene families (Ajayi and Showalter, 2020). The chromosomal distribution and colinearity analysis results indicated that tandem duplications and segmental duplications might contribute to adaptation to the environment for *C. goeringii*. The majority of 22 pairs of collinear genes had *Ka/Ks* values <1, implying that purifying selection may have taken place on these *CgMYB* genes. Therefore, the results show that the evolutionary processes are relatively conservative in *C. goeringii*.

Flower color is an important feature of ornamental plants in the orchid family. The R2R3-MYB (mainly S6 subfamily) gene family has a regulatory role in anthocyanin synthesis, mainly in concert with the BHLH and WD40 genes, which regulate the structural genes responsible for anthocyanin formation. Multiple R2R3-MYB genes are associated with flower color formation in Orchidaceae. For example, the *PeMYB11* gene in *Phalaenopsis* acts mainly on the formation of red spots in the labellum, and the center lobe of the lips’ color is controlled by *PeMYB12* (Hsu et al., 2015). The *RcPAP1* gene in Cattleya activates the anthocyanin biosynthetic pathway to produce the purplish-red color in flowers. At the same time, *RcPAP2* expression encourages lower cyanin accumulation in the perianth, giving it a pale pink appearance (Li et al., 2020). Overexpression of *PmMYBa1* and *PyMYB10* in Prunus and pear contributes to anthocyanin production in Rosaceae (Feng et al., 2010; Zhang Q. et al., 2017). In this study, we selected two genes related to the anthocyanin pathways of *C. goeringii* to analyze. And then, we collected samples from different flower tissues in pale-yellow flowers with purple-red spots and the light purple-red flowers for RT–qPCR. The expression levels of *CgMYB91* peaked in the lips, especially in YF, suggesting that *CgMYB91* is associated with the formation of red spots on the lips of yellow flowers. The expression of *CgMYB32* was different from *CgMYB91*. The expression of *CgMYB32* was low in the lip of YF and highly in the petals of the light purple-red flower. We hypothesize that *CgMYB32* may be more highly expressed in flower organs with low levels of anthocyanin content and have an opposite regulatory effect to *CgMYB91*. Therefore, this study suggested that *CgMYB91* and *CgMYB32* may be related to the anthocyanin pathways of *C. goeringii*.

## Conclusion

This paper identified 104 *CgMYB* genes and performed phylogenetic analysis to classify these genes into 22 subfamilies. Analyzed gene structures and conserved regions and found that the *CgMYB* gene family was subject to gene duplication events and purifying selection pressure during evolution. In this study, two genes that may be associated with anthocyanin formation were targeted and selected for qRT–PCR validation. The results will offer reliable information for further research on the floral color of other orchids.

## Data availability statement

The datasets presented in this study can be found in online repositories. The names of the repository/repositories and accession number(s) can be found in the article/Supplementary material. Sequence data used in the study can be found in Supplementary material. *Cymbidium goeringii* genome data can be found here: NCBI, PRJNA749652.

## Author contributions

S-PC and Z-JL contributed to the conceptualization and designed the research. JC, Y-YB, and D-KL prepared the original draft. JC and Y-YB performed the experiments. JC, Y-YB, and Q-QW analyzed the data. JC, Y-YB, D-KL, Q-QW, XD, and DZ contributed to the visualization. All authors reviewed and approved the submitted version.

## Footnotes

1
https://www.arabidopsis.org

2
https://www.ncbi.nlm.nih.gov

3
http://pfam.xfam.org/

4
https://www.ncbi.nlm.nih.gov/cdd

5
http://smart.embl-heidelberg.de/

6
http://www.expasy.org

7
http://www.csbio.sjtu.edu.cn/bioinf/euk-multi-2/

8
http://evolgenius.info/evolview

9
https://meme-suite.org/

10
http://weblogo.berkeley.edu/logo.cgi

11
https://www.ebi.ac.uk/services

12
http://bioinformatics.psb.ugent.be/webtools/plantcare/html/

## Abbreviations

TFs: transcription factors
MYB: avian myeloblastosis viral oncogene homolog
BHLH: basic helix-loop-helix
WD40: WD-repeat protein
HMM: hidden Markov model
NCBI: national center for biotechnology information
ExPASy: expert protein analysis system
Mw: molecular weight
pI: theoretical isoelectric point values
GRAVY: grand average of hydrophilic
AI: aliphatic index
II: instability index
NJ: neighbor-joining
EMBL-EBI: European Bioinformatics Institute
GO: gene ontology
FPKM: fragments per kilobase of transcript per million fragments mapped
RT-qPCR: real-time reverse transcription quantitative polymerase chain reaction
Trp: tryptophan
YF: pale-yellow flowers with purple-red spots
RF: light red-purple flowers.
