# Supplementary material for: Genome-wide identification and analysis of anthocyanin synthesis-related R2R3-MYB genes in Cymbidium goeringii
Source: Front Plant Sci. 2022 Sep 28;13:1002043. doi: 10.3389/fpls.2022.1002043 (PMC9554442; doi:10.3389/fpls.2022.1002043)
Supplement: Supplementary file 1 [file Table_1.docx]

**Supplementary Material**

**Genome-wide Identification and Analysis of Anthocyanin Synthesis-Related R2R3-MYB Genes in *Cymbidium goeringii***

**Jiating Chen^1,2^, Yuan-Yang Bi^1,2^, Qian-Qian Wang^1,2^, Ding-Kun Liu^2,3^, Diyang Zhang^1,2^, Xiangqing Ding^1,2^, Zhong-Jian Liu^1,2^，Shi-Pin Chen^2,3^***

1 College of Landscape Architecture, Fujian Agriculture and Forestry University, Fuzhou, China；

2 Key Laboratory of National Forestry and Grassland Administration for Orchid Conservation and Utilization at College of Landscape Architecture, Fujian Agriculture and Forestry University, Fuzhou, China;

3 College of Forestry, Fujian Agriculture and Forestry University, Fuzhou, China.

***Correspondence:**

1. mail addresses: fjcsp@126.com (Shi-Pin Chen)

**Supplementary Tables**

**Supplementary Table 1.** CgMYB protein sequences used in the phylogenetic tree

| **Protein sequences** |
| --- |
| >CgMYB1  MGRSPCCDENGLKKGPWTPEEDQILVLYIQKHGHGSWRALPKLAGLNRCGKSCRLRWTNYLRPDIKRGKFSPEEEQSILNLHSILGNKWSAIATHLPGRTDNEIKNFWNTHLKKKLIQMGFDPMTHRPRTDFFAALPHLLALANLVDHRPSTWLADGLSAEASKLQCLQYILNAATPTSADNSMNNLLNSTDQSFPSSLDSMNSNQPLFNTHNTEIDVPLSYDQPLMSNENSGFSLFRHGEISTPVSALPPLTDLSNPGDACSTSSCGGSAAASFWPDLLLDDPFMAEFA |
| >CgMYB2  PEKNTKKKYPKPYPLMAASEEMRKGPWTEQEDAQLVCYVHLFGERRWDFIAKVSGLNRTGKSCRLRWLNYLHPGLKHGRMTPQEEQLIVELHSRWGNRWSRIARRLPGRTDNEIKNYWRTHKRKKAQERQSSLLSPSSSSSSSSSSLNSIVMPDAKNKWLEQINNCSFISMPGFDQEEGELEGYSMDQIWNELAVSEIPWLNFEGCKEKMNDVEIPEMASPLWDYSESQMWKVDGEDLKLVSPMGGRMIPNYKYGTEM |
| >CgMYB3  MGRPPCCDKVGVKKGPWTPEEDIILVSYIQEHGPGNWKAIPNNSGLSRCSKSCRLRWTNYLRPGIKRGSFTEQEENLIIHLQALLGNRWAAIASYLPERTDNDIKNHWNTHMKKKLRRMENEGGMIAGSGFSKNQSIAKGQWEKRLQTDINMAKQALQEALSMEKPSFLYESKPSSSSHSSSTPPSSTTYASSTENISRLLENWMRKAPKNSDKSTQHSVKNFVNIGADSTSSDGTITSVANNHIVSPEISQFQVDSKPCLDERFPLSLIETWLFDENYWGGNGSNNVLDFSLDDASHELF |
| >CgMYB4  MAANDVDRIKGPWSPEEDEMLQMLVEKHGPRNWSLISKSIPGRSGKSCRLRWFNQLSPKVEHRPFTPDEDETIISAHRRFGNKWATIARLLSGRTDNAIKNHWNSTLERKEAAAAAAVAWTSEERMMGALEDCRPIKRSNGATLCFSPGSPSGCDDTGPTKRNYPQPSSLQVGKVIKADAPDSTTGKWNPCDPFTFLTLSLPGSSCGQNESSDNHKQTDPQLLEKEPPSASMASPFSLEFLEALQEIIHQEVKNYMSGLEHRGILPPPLPPEEDSKLNSVAKGIGISRIN |
| >CgMYB5  MHPRQHTTNLPRVTSCKLVLRRGGEEQTSSRKSYEANLRLMVVVSKEIRKGPWTEQEDLQLVCFVSLFGERRWDFIAKVSGLNRTGKSCRLRWFNYLQPGLKHGRMTPQEERLILELHSEWGNRWSRIARRLPGRTDNEIKNYWRTHMRKMAQERTRNSSPSASTISNCLSTHSESTLEATETKKEEDGSPLMAETGSRENVEAVKVYPMEQIWNDIASLESSGGSNFDQCMDERYNLSCDPPLMTSPVHETFVDNLWMTDDHDFSKIATTNDLLFLSSGMVQDSRIS |
| >CgMYB6  MGRAPCCDKNGLKKGPWTPEEDQKLINYIQKHGHGSWRTLPKNAGLSRCGKSCRLRWTNYLRPDIKRGRFSFEEEETIIQLHSILGNKWSAIAAQLPGRTDNEIKNYWNTHIRKRLLRMGIDPVTHFPRLDLLDLSSLLNPMLYNPGQLDLSRLLGIEPLVNSELLRFASNLFQPQCQNTNLVQAQEQQPPNLSTSQQDLVPPYSQLQSLVQSSSQNVHFDSSAQLKWQSNSRAANVRNLGDNFMPVDNNVSYNDQIGQFMLPNFVQDDDQALDFQFPYSLTHDSVLSTPSSSPTLNNSNCSAYLNSSTTEQDQDSYSSTSLFNFEIPDLLDVSDFM |
| >CgMYB7  KHNFFVQMCTRGHWRPEEDEKLKELVARLGPHNWNTIAEMLHGRSGKSCRLRWYNQLDPRINRCPFTEEEEERLLASHRIHGNRWSIIARLFPGRTDNAVKNHWHVIMARRSRERSKLQTKQSSLSLESHLVRKEKKEIMNYEFSKGFLFSSLIGEGRSRTLCAFKHPISQSQEVHSYSPNFHEEGDTSMESSIEFYDFLQVKSDSNETGECSRREDERDQNEKEQQCEAGVPFIDFLAVGS |
| >CgMYB8  MVVTREGIRKGPWTEQEDLQLVCFVSLFGERRWDFIAKVSGLNRTGKSCRLRWVNYLHPGLKRGRITPQEERLILDLHSQWGNRWSRIARRLPGRTDNEVKNYWRTHMRKMAQESKRCPPLSSSTCSSESEQLEGDNGMEKNEQRSYMMPKNLEIKEEYEVKVYPMDQIWNDIAATLEPTTTSGLSFESYGHEAGNVSCVSAAMASPVWENCAESLWKMDDDELCVGIKS |
| >CgMYB9  MAFSFSSAPQFSTVETEGRRNEAVLILEEQRVKRSSNFEGLSEKNGRFEDQEGIELESGHSKLCARGHWRPAEDAKLKDLVAQYGPQNWNLIAEKLEGRSGKSCRLRWFNQLDPRINKKAFTEEEEERLLSAHRLYGNKWALISRLFPGRTDNAVKNHWHVIMARRQREQSNAYRRRKPCTSSKIFHKRMRRNAVTIYSFPQQPQQFEYLFDTQGELVAGRSGCFERLFASAIDMRQASPLIVVPGIHHSGFSDSNSEASASDSAINNAFISEEAELESEKISLPFIDFLGVGVT |
| >CgMYB10  MVRSPSREERGLKKGPWTPEEDQKLIDYIQKQGSHGSWRQLPRIAGLNRCGKSCRLRWTNYLRPDIKRGNFSEEEEKLIINLHSMLGNKWSLISTKLPGRTDNEIKNYWNTHLKKKLLLMGIDPVTHRRRTDLELFANFPKLLSSSTDSCSLINPLMENSLGLQADTVQLARIHLMQVLIQVLLKAASFSSSSSNPNAYSNLVLNNLMSSSLLPLETFSNNSSSSSLPIETSAFGEPSQTPTNYQDFNVQNSENISKQLLFHSNSNDLFASSENHSVDQKQDQIDLNETSAAKSVNARYLDTLNLTDLDDTDLSWKDVLDQIYW |
| >CgMYB11  MGRYPCCDEVGVKKGPWTPEEDQKLVEYIKENGHGSWRHLPKSAGLNRCGKSCRLRWTNYLRPDIKRGKFSEEEERLIIHLHSVLGNKWSSIATRLPGRTDNEIKNYWNTHLKKKLLLMGIDPVTHRPITNIDLIANLPNLLSHTNLKNLAISCDQSLHQLHTDAAQFARIQIIQSLLHILSSPHPAYPADILLQLMNTKLEGLSQLVAGQGLIPNNIEGLKQSSNGFDYQEIASKIMDDISKQERIVF |
| >CgMYB12  MFPILPFYFSFLFYLASKRLPFSLSLSLSLSPSYSFSKARKPKQKSRYMGRVPCCDKDGLKKGPWTPEEDQKLIDYIQKHGHGTWRTLPKNAGLARCGKSCRLRWANYLRPDIKRGRFSFEEEETIIQLHNILGNKWSAIAARLPGRTDNEIKNYWNTHIRKRLLRSGIDPVTHRPRLDLLNLSSLLNTVLFNQSARLDASKLVDIKPHLNAKFLRFAVSLLQSQYQKQNLLKHNLFQQSHPNCHQTITQPLPSSFLYNPVQFNNAGQWQYNEESSCNLSNLNYENMAQSMTGLLHGAFNAEIPSCQNLYSSLDSVLSTSVSSVTLNNSSNIISSNIEDEKDTYCSNFFDFKIPELLDMSEFT |
| >CgMYB13  MENLDAVKHGEMKACPRGHWRPREDEKLRQLVEEFGPQNWNTIAEKLQGRSGKSCRLRWFNQLNPRINRRPFTEEEEESLLTAHKIHGNKWALIARFFPGRTDNAVKNHWHVIMARRQREKAKFLCKNGPSSTWPAFSNFSLTSERRVNYGTLESSRFLNVPLLSFCDAYPACSLSGTGYGGGCGSGMIRNMNEVLRLGCNSRREILSINCRGGIQFLEEGNDEASWRREDLTYIDFLGVGKDC |
| >CgMYB14  MGRSPCCEKVGLKKGPWTPEEDQKLLSYIEEHGHGSWRALPAKAGLQRCGKSCRLRWTNYLRPDIKRGKFSMQEEQTIIQLHALLGNRWSAIATHLPKRTDNEIKNYWNTHLKKQLAKMGIDPVTHKPKSDALASADGHTRSTANLNHMAQWESARLEAEARLVRESKLRSSAPPSPFPPQYLPQPPPPVSMPTAASPSLDVLGAWQGEWPKPVVNSQAGSHNIDLESPTSTLSFSENMLPSRIPGMGTANDSTNWKCLKKPGFSLDTAEAFVNAEATSWLTGSCSGGFAAGFTGMLMGNTNKQNSTEGCDDSDIAGGSCVDVEEGEDEAEENKNYWNSIFNLVNSSSPSNSPPAVF |
| >CgMYB15  MGRSPCCEKVGLKKGPWTPEEDQKLLSYIEEHGHGSWRALPAKAGLQRCGKSCRLRWTNYLRPDIKRGKFSMQEEQTIIQLHALLGNRWSAIATHLPKRTDNEIKNYWNTHLKKQLAKMGIDPVTHKPKSDALASADGHTRSTANLNHMAQWESARLEAEARLVRESKLRSSAPPSPFPPQYLPQPPPPVSMPTAASPSLDVLGAWQGEWPKPVVNSQAGSHNIDLESPTSTLSFSENMLPSRIPGMGTANDSTNWKCLKKPGFSLDTAEAFVNAEATSWLTGSCSGGFAAGFTGMLMGNTNKQNSTEGCDDSDIAGGSCVDVEEGEDEAEENKNYWNSIFNLVNSSSPSNSPPAVF |
| >CgMYB16  MAARCKDVDRIKGPWSPEEDEMLQILVVKHGARNWSLISKSIPGRSGKSCRLRWCNQLSPTVEHRPFTPEEDRTIISAHQRFGNKWATIARLLNGRTDNAIKNHWNSTLKRKYSGAALEDEGRSLKRSNSAGLSFSPGSPSSSDVSDSSHHSQPLMTPPPMVAPVYRPVPRTGCIVPSQLPHAIDTESSGTGSITISDPITSLTLSLPGSASDQSEASDHHLRQNQSDLLLIALPTQPEKLPLPAAAAFPFSNEFLAVMQEMIRQEVRNYLSGMEQRETMIMSPSHESLCNTVIKRIGISKID |
| >CgMYB17  MGRSPCCEKAHTNKGAWTKEEDERLITYIKAHGEGCWRSLPKAAGLLRCGKSCRLRWINYLRPDLKRGNFTVEEDELIIKLHSLLGNKWSLIAGQLPGRTDNEIKNHWNTHIKRKLLNRGIDTQETHHPIAKAATAAPSKPTSMSAANTQQSMAEDEDAHKNWQGLPDLNLELTISLPATSQMPVASRDNSTQSTCLCYSLGFPSISLACSCNMLQS |
| >CgMYB18  MVRPPSPVHPFEQQPPADPLSQPKKISKLNCNSGCSNSTQIFFSICLLLHTARKFARPKYSCMLLQHDEEIRMFFIQAYATLLISAIGAKITRPRYRKTPTMHVSHKRDLKAYVTLLISTGNIQSLSAAALDKYAAHAWLLSPPCQPYTRQGLQKDSADARASSFIRILELIPQMRQPPIMLFVENVIGFEISNTHKHMMGILAQTCFVTQEYILSPLQFRIPYSRPRYFCLAKRKPLCFKNPSLNYKLCHAFTPRIPLEDSTLMDGCLPGKVNEECYLSCLPIRNFLETSVPSVGFEPVCSDKEADVSRDFLEKYAVPHSLIDRWGSAVDIVYPNSKRCCCFTKSYFRYVKGTGSLLATSENSNYEAAKSTGTCFMKDLGLRYFTPRENLASSSPPLPPPSSSPSASIECAGPQAEGFGMKSGAASREDRVKGPWSPEEDAILSRLVAKFGPRNWSLIARGVPGRSGKSCRLRWCNQLDPQVKRKPFTEEEDRIIIAAHSLHGNKWAVIARLLEGRTDNAIKNHWNSTLRRKCIEIESCKRAPCEGQEDISADILEKTKGSSEETQSFGDVKPFTGTEVRDISSRESLSHHSEDRVNTVGPEFKQPPTISRPVPRLSAFSPYNPGFSHATSSLQSRSPLNAPHFRACLSGSGDYKLFENMLWEPQVPSRCGHGCCGTKTRDQNTSSSLLGPEFVEFIEPPPILNREIASMASELSNIAWLKSGLQTGIYSSCQMNPSSSSCTASTC |
| >CgMYB19  MGRPPCCDKIGVKKGPWTPEEDIILVSYIQEHGPGNWRSVPTNTGLSRCSKSCRLRWTNYLRPGIKRGNFTDQEEKLIIHLQALLGNRWAAIASYLPERTDNDIKNYWNTHLKKKLIRKLEIDVETEGESSLKRRASISNYQSVTKGQWERRLQTDINMAKQALQEALSMEKPSFLYEIKPSCSSHSSNNSRSPSSTYASSTENISRLLQNWLRKPPNSSSSSDRANSAESTQISVTANSSTSVGKTVSFGNDKRSSIVSPETCSLFQVESKPSLEPELQFSQLETWLFDENYVGGTESSMNMLDLALDESELF |
| >CgMYB20  MGRSPCCDEVGVKKGPWTPEEDEKLVEHIKKHGHGSWRHLPRNAGLNRCGKSCRLRWTNYLRPDIKRGKFSEEEEGLIIHLHSMLGNKWSLISTKLPGRTDNEIKNHWNTHLKKKLLIMGIDPVTHRRRADLEFLANLPNLLSSSNSCNRMTNSWGSALQLQAADATNLAQVQILQGLLQVLIASSSNSNSSSNLNMTNLLGSSLAPLRNIADILQTARGLTEWLLWSAHGSLPATTQMPNNYQTLPHQDQPEMNGNENQNLLFHSNAPSLVSASPENTPSVSLKQEHINSNEISAANSESSTPFDPWDALSLSDPNGADLGWKEIL |
| >CgMYB21  MGRSPCCDKAHTNKGAWTKEEDQRLVSYIQAHGEGCWRSLPKAAGLLRCGKSCRLRWINYLRPDLKRGNFTEEEDELIIKLHTFLGNKWSLIAGRLPGRTDNEIKNYWNTHIKRKLLSRGIDTQTHHPITATSAAFLSSPEIYSAVPESAVTSDHKCHSGSSSGGGSGNCADLNLELSISLPYYSSPQPSPYSDDASTAAAVRTPETCFCYRLGFRGGEACVCESLRSRLFQD |
| >CgMYB22  MDGGGGGEEKIKGSWSPEEDALLMKLVERHGARNWTLISAGIQGRSGKSCRLRWCNQLSPEVHHRPFTAAEDAIIVAAHGKYGNKWATIARLLPGRTDNAIKNHWNSTLRRRRRVEAATVSALSGPESESDSGGKRPCRRDDLILKVVEPMTPEAVDPATSLSLSPPGESSAAVAGSAATAPDAGTGGGWKEKGWLGNACVMSVMRKMIAEEVRRYMSGLRLDGGGGFYSTVKAESASNGQD |
| >CgMYB23  METEKQNAIVEVELRRGPWTLEEDLILTNYISKHGEGRWNTLAKCAGLKRTGKSCRLRWLNYLRPDVRRGNITAEEQLLILELHSRWGNRWSKIAQHLPGRTDNEIKNFWRTRMQKHANQQCMPPLLERIRTVVDSSRAVMGVHTASASSSGRDGFSSSSATGNYDDHLDLKDMASTLEEEGWPEFPAHGCEGSMLGFWEFSAMGPNMYA |
| >CgMYB24  MAADKPEESRLCPRGHWRPGEDEKLRQLVQEFGPQNWNSIAEKLQGRSGKSCRLRWFNQLDPRINRRPFTEVEEERLLAAHRFHGNKWALIARLFPGRTDNAVKNHWHVIMARRQREKTKFLIDKSSFQQNHSHSHILNQQTCCSISPNQFNHYKYNHGILQSSHQPLQMFSNSSNIYWRFGMLDGHMGCCRTVSGGVNQEEKDNDEALRSKDVAYIDFLGVGKD |
| >CgMYB25MGRSPCCEKAHTNKGAWTKEEDERLVAYIKVHGEGCWRSLPKAAGLLRCGKSCRLRWINYLRPDLKRGNFTEEEDELIIKLHSLLGNKWSLIAGRLQGRTDNEIKNYWNTHIRRKLLSRGIDPATHRPLQGPADVSFVKADEKGASLIRKEDEQKSNNSSSSNSNSRSSSDEPKRWRCPDLNLELGISPPSHQEEDSEEDLMREELGLCFRNGLLEFR |
| >CgMYB26MGRSPCCEKAHTNKGAWTKEEDQRLIAYIRAYGEGSWRSLPTSAGLLRCGKSCRLRWINYLRPDLKRGNFTEEEDELIIKLHGLFGNKWSLIASRLPGRTDNEIKNYWNTRIKRKLLDHGIDPQTHRPINSATRPPVLVNPTADRFATTVITKLDERSHSEGSSSASCSIDLNLDLSIVSLPPILRSTIAH |
| >CgMYB27  MGRSPCCEKAHTNKGAWTKEEDERLIAYIRANGEGCWRSLPKAASLLRCGKSCRLRWINYLRPDLKRGNFTEEEDELIIKLHSLLGNKWSLIAARLPGRTDNEIKNYWNTHIKRKLISRGIAPRPTALSMRHKRTTRYPSLKEKTKLWSLSREKTKETRAAVAAVAAAVIAAKSC |
| >CgMYB28  MGRSPCCDEVGVKKGPWTPEEDEKLVEHIKKHGHGSWRHLPRNAGLNRCGKSCRLRWTNYLRPDIKRGKFSEEEEGLIIHLHSMLGNKWSLISTKLPGRTDNEIKNHWNTHLKKKLLIMGIDPVTHRRRADLEFLANLPNLLSSSNSCNRMTNSWGSALQLQAADATNLAQVQILQGLLQVLIASSSNSNSSSNLNMTNLLGSSLAPLRNIADILQVSRQLEALQNGSFGPAHGSLPATTQMPNNYQTLPHQDQPEMNGNENQNLLFHSNAPSLVSASPENTPSVSLKQEHINSNEISAANSESSTPFDPWDALSLSDPNGADLGWKEILEQISW |
| >CgMYB29  MGSLQQRSEAPVLLLSSLWPQASFLTASESPSEREFVRSKESWSFNTSHDDLRLSDESMESRDLETKQSKLCSRGHWRPTEDAKLKDLVAQLGPQNWNLIAQNLKGRSGKSCRLRWFNQLDPRINRKAFTSEEEERLLSVHHHYGNKWSLIARFFPGRTDNAVKNHWHVIMARKQREHSISYRRRRFSSSTSFNPSPHPQTMEHNSSSGESTITNTREESISSATTRTVHGFVLSPHQKKAINFIMGSVRNGSYEKPDDSFSASLVGAKSIGDQITSSDSTSKASAREAVAFSGSELDRERINPPFFDFLGVGAAEEKPLPVCVMPRERERES |
| >CgMYB30  ELREGREKGLRSMGRSPCCEKAHTNKGAWTKEEDERLTAHIKAHGEGCWRSLPKAAGLLRCGKSCRLRWINYLRPDLKRGNFTEEEDELIIKLHSLLGNKWSLIAARLPGRTDNEIKNYWNTHIRRKLMSSGIDPATHRPLHEQQAKTTISFIKGEEKVEEFEGREEGKKSSSNSSEEMSAWLRRRPEEENKQWRCPDLNLELCMSPPPSLKQQALCQEPVKSEELSLCFNCRLGVNKSSECKCGNGFLGLRSGVLDYRRLETN |
| >CgMYB31  MVRTACCDEMGLKKGPWTPEEDRILVSYIQRYGHGNWRALPKQAGLSRCGKSCRLRWTNYLRPDVKRGDFSKEEEETIIYLHAMLGNRWSAIAAKLPGRTDNEIKNVWHTHLKKRLNPNQAMKDSKRRNRNSDMNKEMKKKDMEEQVSPACSRSEASSFVIATENCDVWWKEDSMDFLKELPELDEILTSDAQSASQNNWAGEEEEEDSTGKNYDRTRSSNEEDIRFWMNLLAEAGNLECFK |
| >CgMYB32  AFERRKFTNLGRGTEMGRSPCCEKAHTNKGAWTKEEDERLIAHIRAHGEGSWRSLPKAAGLLRCGKSCRLRWINYLRPDLKRGNFTDDEDELIIKLHSLLGNKWSLIAGRLPGRTDNEIKNYWNTHIRRKLLNRGIDPATHRPISAASTSNLSITDKRNGVNRVSALLPRCPDLNLDLCISPPLEEQLQQDLLEESNCGGEFLRLGSSFLLDYRSLEMK |
| >CgMYB33  MGRPPCCEKEGVKKGPWTPEEDIILVSYIQEHGPGNWRAVPTNTGLMRCSKSCRLRWTNYLRPGIKRGNFTDQEEKLIIHLQALLGNRWAAIASYLPERTDNDIKNYWNTHLKKKLKKLQLSGEDGGGGRTVNPISNNHHQSFSKGQWERRLQTDIHMAKQALSEALSLDTMNLRSPSPPSLGPANACTYASSTENISRLLENWMKSTPKSRSRTSLGSMSNVCSDSTTMESTTPTPAGQIMTSSAVQLGFRPAKIEPNGVLKEESKESSVVAPPPLSFIENWLFDDLSVGNGGDDHQDLVDLPLSDASELFQD |
| >CgMYB34  MGRSPCCEKAHTNKGAWTKEEDQRLIAYIRAYGEGSWRSLPTSAGLLRCGKSCRLRWINYLRPDLKRGNFTEEEDELIIKLHGLFGNKWSLIASRLPGRTDNEIKNYWNTRIKRKLLDHGIDPQTHRPINSATRPPVLVNPTADRFATTVITKLDERSHSEGSSSASCSIDLNLDLSIVSLPHSSVDDSPLTHSVSTSTMTTKMAATPAICLCYSLGFRGSEECGCQAVQEQQAVIRYCSSIEIELRNYFS |
| >CgMYB35  MDGGSNEPFSPFKQMVQKPSWGFGTVLSEEMETKEHGRLNSKLCVRGHWRPAEDAKLKELVSRYGPQNWNLIAEKLDGRSGKSCRLRWFNQLDPRINRKAFTEEEEERLLSAHKFYGNKWALISRLFPGRTDNAVKNQWHVMMARRQRELSDACKRRKSSSSCFITSVKMEEKININSNSSNECTSSRNFHLSLFLNSYKFPGTSVLIGSMESLKLRHRNSGRP |
| >CgMYB36  MVFLSGGTGSFVVEKNIHYMELEKRENETRLSKLCSRGHWRPAEDAKLRKLVALYGPQNWNIIAEKLDGRSGKSCRLRWFNQLDPRINRNAFTKEEEQKLLSAHKLYGNKWALIARLLPWRTDNAVKNHWHVIMARRRREKSDACRKRKQSNSSCIRNSCVSGSTITSNKDEYVSACTDLSLSSSFTSTSFLTSYKLSHLPLKENPIGYLIGSDGKLVTGRADQSDNPDREFESSLTEPLINQNVNSFSLNKTDHKKEKIKLSFIDFLGVGAK |
| >CgMYB37  MSFEKKGVVGALLGTRNQPVQWGVLSHMYINLHTRVYKGGWEVKEEQEEELGQSQAGGEVNLSFFTSIFSSTSNFPFPLSMALHHCSGTAHGMEKRFIEVQSQQVDSHSASPSSPKSFFSQMVSLSINPSSSHSAHHFFPFAANSDANEVFGGKHNMVVAEEEISRDGGGENTNDSSGQSKLCARGHWRPAEDSKLKELVAIYGPQNWNLIAEKLEGRSGKSCRLRWFNQLDPRINRSAFTEEEEEKLMAAHRLYGNKWAMIARLFPGRTDNAVKNHWHVIMARKYREQSTAYRRRKLSQAMHLRFEDNTTASRPLPPFYFSSSMLSPYLIPNGGMSSIKQKPSDFIISDHRDQERTDIFRQNDNEVQALQSSSYSHQASILLTMHQSAGHLPHNISVASSSSGETPAHSAEVEMKTRADNVN |
| >CgMYB38  MKGCLRGHWRPGEDEKLRQLVEEFGPQNWNTIAEKLQGRSGKSCRLRWFNQLDPRINRRPFTEEEQERLVTAHRIHGNKWALIARLFPGRTDNAVKNHWHVIMARRQRERAKFLCKNGSSSTWPVFYNFSLTSERTVNYGTLESSRYLNKPLLSFCDAYRACSLSGTGFGAGSGSDVAEINSNDLQSTNGLRHRVQNVEIGQNINIVRLDEQPIKGSSRQRACMHIEVRRTPMRIKRSPFVKLIASLASKRRNLSNKTIRTQFRRVCAKIERVKVATRILS |
| >CgMYB39  LCLLKSKASSIVSSIHTKDKTRTLMVVMKEGTRKGPWTEQEDLQLVCCVSLFGDRRWDFIARISGLKRTGKSCRLRWVNYLHPGLKHGLMTPEEEHLILELHSKWGNKWSQIAGKLPGRTDNEIKNYWRTHMRKMAQEMKRNSQHSSSSSSTSSSCLSLETSTNGMENQQEVSFLLTMPEEKEEEVKVKAYPMDQLWNEIVTSDSTASGLSFKQNISMECSPVWEHSVESLWKEDDSEEPNLTHDSELMVFNYQNDTKS |
| >CgMYB40  MVVTSPSYQLERVEWHLPDRVLRQFGLIARVDVALVNPSLKWVDGQGKADVDWVDYHQSHIAAWERWRDCLVEGLDEDVDRKTAISQYMCWFRSWASMYLLAAPTDPPTTYYVRALVECIYAEHIVATRDIFQPYFCVVDDHPLQGCVDRYELICKDLDELVHISDTYYGSVPTEDLPRYLIDKQWIEPTPTDFLFLLLLPVAIYARFHVMSSSILLQLPVPPRKQSSSSLPLSSFLLRAPKPKHKSRYMGRVPCCDKNGLKKGPWTPEEDQKLIDYIQKHGHGTWRTLPNNAGLLRCGKCRLRWANYLRPDIKRGRFSFEEEEAIIQLHSILGNKWSAIAARLPGRTDNEIKNYWNTNIRKRLLRSGIDPVTHRPRLDLLNLSSLLSTVLFTQPSRLDALKLLDNKPQLNTEFLRIAANLLMSQCLNENLFEHNQVQESQPTYHQTIPQQLSASLLYNPVEFSNTSQCQYNEASSCSLSDNLVTTNNDLRYEASNVELPSDQNLYSDLDSVLSTPVSSMTMNDSSTAFNNSIEEEKDSYHNDFFDFQISDMLDINEFI |
| >CgMYB41  MGRSPCCEKVGLKKGPWTPEEDQKLLSYIEEHGHGSWRALPAKAGLQRCGKSCRLRWTNYLRPDIKRGKFSMQEEQTIIQLHALLGNRWSAIATHLPKRTDNEIKNYWNTHLKKQLAKMGIDPVTHKPKSDALASADGHTRSTANLNHMAQWESARLEAEARLVRESKLRSSAPPSPFPPQYLPQPPPPVSMPTAASPSLDVLGAWQGEWPKPVVNSQAGSHNIDLESPTSTLSFSENMLPSRIPGMGTANDSTNWKCLKKPGFSLDTAEAFVNAEATSWLTGSCSGGFAAGFTGMLMAAWMWKKVKMKRRRTRTTGTAYSTWSIRHLPQIRRQLCSSNLNIST |
| >CgMYB42  MGRSPCCEKVGLKKGPWTPEEDQKLLSYIEEHGHGSWRALPAKAGLQRCGKSCRLRWTNYLRPDIKRGKFSMQEEQTIIQLHALLGNRWSAIATHLPKRTDNEIKNYWNTHLKKQLAKMGIDPVTHKPKSDALASADGHTRSTANLNHMAQWESARLEAEARLVRESKLRSSAPPSPFPPQYLPQPPPPVSMPTAASPSLDVLGAWQGEWPKPVVNSQAGSHNIDLESPTSTLSFSENMLPSRIPGMGTANDSTNWKCLKKPGFSLDTAEAFVNAEATSWLTGSCSGGFAAGFTGMLMAAWMWKKVKMKRRRTRTTGTAYSTWSIRHLPQIRRQLCSSNLNIST |
| >CgMYB43  MRRERACLPKEELRRGAWTEHEDKLLSDYITSHGLGRWRSLPEKAGLNRCGKSCRLRWLNYLRPGIKRGNITDEEEELIIRLHKLVGNRWSLIAGRIPGRTDNEIKNYWNSYIKKKVAMDVSESHSSSKAATTTTVAAATTTASTTQLSFKSPCNEKANEASSVTSMVKSKTYETEDLNTNRSVLHEQHSSMKPEGHQQVCLPPSASSDFDSGFEFSFGELSNSSVFSCYGWRDAKSMACSSVIGEDCSSRRMEER |
| >CgMYB44  MAPQPLSFTDHGLGNRYIEAQAQEEANSPSRSSSKSFLSHMVSLPINPSCSFSTHHLFHFMGGSDTNEVLEEDNNRADAEEENVNDCTGQSNLCVRGHWRPEEDCKLKALVSIYGPQNWNLIAENLEGRSGKSCRLRWFNQLDPTINRSAFTEEEEEKLMAAHRLYGNKWAMIARLFPGRTDNAVKNHWHVIMARKYREQSTAYRRRKLNQAMDRRSEEIIPNHPFSFSSSIADCSTYLHLLPNDGKNSPLSHAGSYTREKLFDFLSGQNPSNGDKTKNFITRTWEGQRDHNEALSSFYCHRASNLFAMTASSSSGEGGASTVQVDQVNRKEGHGHMVASMSSSPTFIDFLGVGATT |
| >CgMYB45  MVSHSCCLKQKLRKGLWSPEEDEKLFNHIIRFGVGCWSSVPKRAGLQRCGKSCRLRWINYLRPDLKRGSFSQEEEDLIISLHEILGNRWSQIATHLPGRTDNEIKNFWNSCLKKKLRQNGLDPSTHKPLNEEQNEDKSHNLNDSNQMNSAMRPVFDPFPALDFQPGTVLNTYDQLQQPPLIPASDSLNASENYFYGDSSASCINWNCSIGAELNFSSDSSYYYSSFQMKSSMFQNPSDACLDIPQSEFGEDLL |
| >CgMYB46  TWGAPLKSEHVCLEISELRFQKAKLTESLKMRMMGCRGSEKGKVKCKKGLWSPEEDQRLRDYIHNQGHGCWTEVPVKAGLERNGKSCRLRWINYLRPGIKRGRFSEEEEELAMKLHAMLGNKWSQIAMHLPGRTDNEVKNYWNTYLKKRVKQSEGSDSAEVAENPSSDSSAKNHVNNKEGNLDKISLFDPFDSAESSSHSHGYCQEKLQNSWPKVMFADWIFGENSDGLLSSESHDGGKGLTWDSCSFDRREMLSNGDLRAMSHQEFGEGGSYGSCFEPVDLVGGDSFDLLSFGGNFGGFEMNENLMF |
| >CgMYB47  MHLCFIRAHNMTCSENDGDTLMLLKEQLESSSTDEGSCCGSSTTGGGRVTLKKGPWTTAEDALLISYVRKHGEGNWNAVQRHSGLLRCGKSCRLRWANHLRPHLKKGAFSPEEEQFIIQMHSKMGNRWARMAALLPGRTDNEIKNYWNTRVKRRSRAGLPLYPPKLSLQTSNENQLIQSASELSSGNHQNIGVLQGTMNGTLEFRCHPHKANSGPLPSPPRFPDIPVTALPCHGLGYYTRSFLNSGANHFKQTWESETFSPGCHVNQNSIVAPAFEELPSMNSETVYLNPGLGYPYDPDPENKNLASFICSTPGNPGNLSSSRLLPGAVKMELPSLQYPEADYIDWTSSSLLKSSYDAVDMYIQSPENVSVHSDCASPRNKGLLEDLVQESHAISSGKKQSFEKSFDRAVAAVMASGTVKWEEIRDPTSPFCLSAVSLFNDCNPTIISTFDESSACETPMGDKQDKYTPNLPYFLRPDALLGSGWLMGNSRDAEDSIAGALDENLHKERAPVPVPVPAGISSMASLDAYPWTNMPRVCQMSEH |
| >CgMYB48  MGRIPCCEKDNVKRGQWTPEEDNKLSSYIAQHGTRNWRLIPKNAGLQRCGKSCRLRWTNYLRPDLKHGEFSETEEQAIVKLHSVVGNRWSLIAAQLPGRTDNDVKNHWNTKLKKKLSGMGIDPVTHKHFSHLMAEIATTLAPPQVAHLAEAALGCFKDEMLHLLTKRRIDFSSTYAVGMYPTPVPPPHSGEPNLVSDINGEETIQKIKMGLSRAVMQEPNPVKGWGSTAYGEPDNHNLMCEMYPMAIDQGYMYGDQSAYVNDGEGSTWSTCNGGGGGSAAAQQHETGSRGYLLKGEEDDAEESEGVKGGSKEDAGVFGSECVLWDLSEDLMNHIV |
| >CgMYB49  MGKQSCLHKQKLKKGLWSPEEDEKLLKHIAKYGHSSWSSVPKLAGLQRCGKSCRLRWINYLRPDLKRGAFLQEEEDLIIELHAALGNRWSQIAAKLPGRTDNEIKNLWNSCIKKKLKQKGIDPNTHKPLSENEGTEDDKISSGTGNLKQTELDSALELMPGIEKDSTMNSIGHYSLSQLSCSTSTTILSGSSNLLLWLKQSNREFGMNPELSCSSITSATPSVSSSNLYNSMDLKAIADLPYWEAGNPNSSSNKNCTSLNGDLCSWPDLTAERGNKYHLTGGNEEMKWSECLQSNLPVSSLPHCHNQPLHAGDLKVETHFDLESINLCFQNH |
| >CgMYB50  FENHFCSPIGRLPALVGMGRHSCCHKQKLRKGLWSPEEDEKLLKHITKYGHGCWSSVPKLAGLQRCGKSCRLRWRNYLRPDLKRGTFSQQEENLIIELHAVLGNRWSQIAAQLPGRTDNEIKNLWNSCIKKKLRQRGIDPNTHKSLSETENPDENKVSTSSERNSILYTGDSLKQTVPQLTSSLMQSLDKNSIPVKEFCIDNATTCLRSSSNSISSFPFPQLNYGTSATASLPLSSNPFLWFNQNCRVFDMNPELSCTSMSTMGPSISSSVLTNSIDLKSPVNLSSSCLPPTCTGAPIFSYLDAGNPSRCSSGSSSSSSFFDSGFLSWPDLTTEKEIQINFQGDPEELKWSEYLQGNFPVSSVLHSHNQPLYGGDANSESHFDIEGVGLWHQNQQPQHQQHQQQPQQQASDLYGKEYQRISTLFGQI |
| >CgMYB51  MEVQYGWGTFEDGWRKGPWTPQEDKLLTEHVKLHGEGRWNCVSRLTGLKRSGKSCRLRWVNYLRPDLKRGKITPHEETIILELQAKWGNRWSTIARSLPGRTDNEIKNYWRTHFKKGKMATNIERARAKFLKQQQEQKQQQQRQQLQLEVPQFEEIAITQLMQEIAYMCNMPYVFQGEEFTRKSMMSSDNRFGDGEAYYATWENLWNLDDLGLDMGDQALSFYY |
| >CgMYB52  LLSWIGAARYKDLGFLASDLFPPREMGRAPCCAKQGLKKGPWTPEEDKILVDYIQSNGHGSWCSLPKLAGLLRCGKSCRLRWTNYLRPDIKRGPFTAEEQKTIIQLHGIVGNKWSTIASQLPGRTDNEIKNYWNTHLKKRLLRMGINPDTYAPASPSSSAGGGGGAGFPVTRHMAQWESARLEAEARLSRESLLFSSASSAAAVSAADSEPADDSLPAARKPESDFFLRIWNSEIGDAFRKPLPSALPRSAAEEATTAPTAESKSCASAGGEAPSRRTRRAPTNWTRRRTRRTSST |
| >CgMYB53  MGRSPCCSKVGLHRGPWTAREDALLTSYIQNHGEGNWRSLPKLAGLLRCGKSCRLRWMNYLRPDIKRGNIGPEEEDLIIRLHRLLGNRWSLIAGRLPGRTDNEIKNYWNSHLSKKLKKQGLKLREATPRTKQSNSSSNNKKQSTKIINQSETLGGTNETKIYAPKPTRLTSRIHVMENSSEEEKGSSDEMELSANYLSDGREHDFLNTNQYLDLGFDQFLPLQQDSVMFERLYDEYSQLLQSEVNDVNLFAYDQYLMV |
| >CgMYB54  RMGRHSCCYKQKLRKGLWSPEEDEKLLEHITKYGHGCWSSVPKLAGLQRCGKSCRLRWINYLRPDLKRGTFSQEEENLIIELHGVLGNRWSQIAAQLPGRTDNEIKNLWNSCIKKKLRQRGIDPNTHKPLPEIEEQREKIAEIDSSGHPTPEPAKQTEQPAETAVLNNFVRPANSSAASYYSLPNLTYSHECGNNIGLIQQFWFNQSSKFFNTNNPNSEFSFNSVSSLLPSVPRSTLSTSKELKPLTNLLMENSPSGFYWEAGNSSNSSASSGSNDPLFDGSIFPWTQLMPERDTNIQLHGETEDLKWSEYLNGSIPLSTDLQ |
| >CgMYB55  MGRAPCCDKANVKRGPWSPEEDEALRSYIQNHGSGGNWIALPKKAGLKRCGKSCRLRWLNYLRPDIKHGCFTKEEDDMIFTLYSKIGSRWSVIASKLPGRTDNDVKNHWNTKLKKRMMETQARLSGSHYRSSAPPLMASEPLALTDHSLASINCASIRPTDQIESQSFNGSFDEPLDLRVPRQNPSFLDLDFGSITDLLCSSTYEETIETIWADASEEIKLGELSQSVAYLY |
| >CgMYB56  RKRRRRLKNKKKREMGRAPCCDKANVKKGPWSPEEDAKLKSYIEKHGTGGNWIALPQKIGMKRCGKSCRLRWLNYLRPNLKHGGFSEEEDNIICSLYVTVGSRWSLIASHLPGRTDNDIKNHWNTRLKKKLLGKSRKDHQHHSRRIPKQLEPKKSEETNDFYWLDPALPPYPETDSQASIKKVLMKLGGRISIDEEAEIPPLINAPDHNSIYCSSNISLMASSAQLSSFNECLSTNLNDIFASNEVKLEEFDNYSLYGMTTMAEEITGLLGMPEVVNWSTSDVSSSIYSPMASTAVSYHGSMQQYVYQDHQMHLGMELSFKYE |
| >CgMYB57  MGRAPCCEKVGLKKGRWTAEEDQLLVKYIKTNGEGSWRSLPKKAGLLRCGKSCRLRWINYLREDLKRGNISKEEEDTIIQLRANLGNRWSVIAGHLPGRTDNEIKNYWNSHLSKKVEGLHDKEDENCDATGVIKVRKRKGGRPSKSAKKKKAEVVISSMDEGTKLKDVMEASKSEQGESIVTDSEPQELSLEDLPSIEILLVDEENIEGGRALNPNNEEEGKRKEDSGVIVEVVSNEEGGSSFDEIEKLVDWELEELALRLWEEGEGVGEVTPWVWEDESPRINGEFGDFFDYEKFDAFKEDDYLDDWLLSDASECSSWFSSCG |
| >CgMYB58  MGRAPCCDKASVKKGPWSPEEDERLKSYIEKHGTGGNWIALPHKIGLKRCGKSCRLRWLNYLRPNIKHGSFSDEEDSIICSLYVAIGSRWSIIAAHLPGRTDNDIKNHWNTKLKTRLLGKQRKNQQNRRSCNLKQVMKKNKADKDQSASATTEANKAWLNLPISTITFHSTDHASYDNQSQTSISKLQMKLADVGLPCCGNGPHQVQVAPSLRIPQAIYETPLNTMAYSSHENTLNDGSPLKNVGPEYFEQGFNYSNNNSLMKLEGFDFFYGMSSFINESINVSESITWSEMNSFTTSVLPISSTNEETLDFTSWETVKQC |
| >CgMYB59  MGRAPCCDKENVKKGPWSPEEDVKLKSYIEQYGTGGNWIALPQKVGLKRCGKSCRLRWLNYLRPNLKHGAFSEEEDNIICSLYIRIGSRWSLIAAQLPGRTDNDIKNYWNTKLKKKLLGKSSHKNHHQSQYTRRAPKQDPSNLQEIINTVEISNGSTSSTSYWIQPATPTYMSSSTEIKRLLPKLEGVPPLITPDHSDLNDTNSYILEASPQPSSFTNCLSSSSTELDDIFGHFDSFYGSNGMSGEITGLLSMPDQVFNSLMYPPMAVAAKASYQGTIGANGRIV |
| >CgMYB60  MGRAPCCSKVGLHRGPWTSREDALLTSYIKNHGAGSWKSLPKRAGLLRCGKSCRLRWMNYIRPDIKRGNISPEEEDLIIRLHSLLGNRWSLIAGRLPGRTDNEIKNYWNGHLSKKLRNQGFAMREAVSRRPKPSRDHSSMKKTNNTTTEMKSTSEKATVSKIYAPKPTRIERRYRNYVMSAVTESNEETEGSDGRLDSGEESSSGNYQENISCSDNGEEANLLDVNRYLGLDLDSENGDFFLQDHEVVERVYQEYLQLLNVETENEKQII |
| >CgMYB61  MGIRPPCCDKLNVKKGLWTAEEDAKLLAYVSTHGSGNWTNVPKKAGLKRCGKSCRLRWTNYLRPNLKQEGFSSQEDELIVTLHATIGSRWSIIANQLPGRTDNDVKNYWNTKLSKKLALNGIDPVTHRPISEIKHSITTLHFAAATAAGQPLPSPAATVGVSLHDSRLISINRDLKKILLSPPIPPPLEPATPPVHWNAAEQTAAAPRSFPPPPPPPPQDLEWVQFLAEDAFLCIDEHDAYRAPPFATVDADFDGMEEEADVSSFIDEMLDRDREIISEFSGLVGGHYAL |
| >CgMYB62  MGRAPCCDKANVKRGPWSPEEDAALRSYVERHGTGGNWISLPQKAGLKRCGKSCRLRWLNYLRPDIKHGGFTEEEDNIIFSLYKTIGSRWSVIASHLHGRTDNDVKNYWNTKLMKKIMAAGETINSPISNNQNTNKFHQPSPSPPATSPPVQIPVMKAEAFLPPTTLDSNFPYAFEPYQLPQLPAELAVTGYNLCPPSEDPSLSVSASSSSITVDDGGTNSYIDWNGEGAYFDQAAAFFPELGFSSWTELLGGFVTSGTYESPTSLWANTDTKPQGVHQSLYS |
| >CgMYB63  MGRPPCCDKPGIKKGPWTPEEDLILISYIQEHGPGNWRSVPINTDMTAASNVIAGLLRCSKSCRLRWTNYLRPGIKRGSFTPYEEGIIIHLQALLGNKWAAIASYLPQRTDNDIKNYWNTHLKKKLIKLQETIGSHNPLAIEAATVNTEFAGVQLQQQHPQYASCTYASSTDNISRLLEGWMRSSEKNTMQSSSIDIKNSGDSLICHEEFYHEKLPEMMPVAAEAVVEVEQNRSLTFLEKWLFDDGTGHVDGFMELPSDQLI |
| >CgMYB64  MGRQPCCDKLKVKKGPWTAEEDKKLVAFILNNGHCCWRAVPKLAGLLRCGKSCRLRWTNYLRPDLKRGLLTDAEEKIVIDLHASLGNRWSKIASKLPGRTDNEIKNHWNTHIKKKLLKMGIDPVTHKLLNDQTSSTAMASPNSTVTDDEKFSKKHEPLDAEKAIINTVSQEESTNPLQNCSNAIDRDEQVIGCLWDDDMPFIDKLWSSPISNEAWERSSEWLLDYQEFGIGDLELGRVESIGFADEQIKLN |
| >CgMYB65  FVPTYASHHISLLFLRVPFNPYKGLHTFLRSERQREREREMGRAPCCDKANVKKGPWSPEEDAKLKSYIEEHGTGGNWIALPHKIGLKRCGKSCRLRWLNYLRPNIKHGGFSEEEDQIICNLFVSIGSRWSIIAAQLPGRTDNDIKNYWNTRLKKKLLGRRRESSSTSQHRHLSDDTDCNTKPNPNGSTQILTASALERLQLHMQIQGLHCPFSFYNNTSLWPKLYPLGNDCKIFQPLSTDATATAVHPLKLSQQAMEPIEQANISNSMHPNIQGRLGASSSSSSNLDIELHDLLYGKESKLFSEIDSFNDLNIEEGTDWLGSNGFEEKSSSSSWESAFALHPDSVLQEYGLGYDL |
| >CgMYB66  MEGQYYGWGTIADGWRKGPWTPQEDQLLIEHVKLHGEGRWNSVSWLTGLKRSGKSSRLRWVNYLRPDLKRGKITAKEEAIILELHARWGNRWSMIARSLPGRTDNEIKNYWRTHFKKSTKNVEKTRAKYLRLQQDQKQQLLLCRSNIMKEETSSQTQMAMMPKEMEEMTFLCNNMPLMLHEGGFVGESTTSSEDRTSEEEAYYDTWGRLWNLDDMGDPTLTF |
| >CgMYB67  MEFSPPPPPPLFPSERDGKTNETGFLLEQQQQPSLTGWGLEGFSGGSGSYGSDKNGQSEEHDGEHESGQSKLCARGHWRPAEDAKLRELVAQYGPQNWNLIAENLEGRSEQGLTRENVLPFSVLLFFYSIDMVVGKSCRLRWFNQLDPRINKTAFTEEEEERLLSAHRLYGNKWALIARFFPGRTDNAVKNHWHVIIARKHRELSNSHRRRKASSSISSSAQAFDKNLQVNCYNNACIAESTISRNIDESFSTCTDLSLNSYAYRSFPGDGFFTRHIHEKQPQQFQYLTGSDEKAVTGRYGYSQRPFDSENDFRQVSPLVFVPGTYQSGYSDSNPEASATGSVVKKGNAFIVEEGDHDREKISLPFIDFLGVRAT |
| >CgMYB68  MAKNPVSSRSTFPLFPIPVCRSLLRSAHRSGREISATEAPPAKGSSSPARRGRRRPYFGQTNRKPQFYFSFPPKRRLLRSSAGFHMIPPYGLYHGYSQQPSEQAEPEDQQLEHQLRDRPRRPPAHYTPIVMRYRIVGIIGDRPVVPEEDQKLIDYIQKHGHGTWRTLPRNAGLARCGKSCRLRWANYLRPDIKRGRFSFEEEETIIQLHSILGNKWSAIAVRLPGRTDNEIKNYWNTHIRKRLLRSGIDPVTHRPRLDLLNLSSLLNTVLFNQPAQLDVSKLLDIQPQLNTEFLRIAVNLLQSQCQNQNLLKYNQVQDSQPICHQTTPQQLHASLLYNPAQFFDASQWQYNEASSCSLSETLVTSNNNVNYENMEKPISSLLLEASNAEIPTDHNLYSNLDSVLSTPASSVTLNNSSRIINRSTEDEKDTYCSSFYDFQIPDLLDMTAFTSFDDFLLPSSSRLKSTVTFGDDSGSSISFEEKFSLADSTFFCALPVVLCKHKMLVLYVEVEEVNVASTSTDPIIRSVGIPLTTHQDFIAPAPLGTQHSFDELIPSYVAEEHHPSARRRRRRPAREASPPLEQAPTEVHPSIAPLRGRHRAAHPDTSTVLEAVSHLLMLKEWPITCRRFICGLEQVGLHYVSQLHFIRLDHHLLTALVERWSPQTNTFHLTIGEMTVTLQDVAMILGL |
| >CgMYB69  MRPPTVVKKERASTTAVASGGAAAAPQQLLKKGPWTAAEDAILKEYVRKHGEGNWNALQKKSGLQRCGKSCRLRWVNHFRPNLKKESFSPEEETLIIRLHAQFGNKWAHMAKYLRGRTDNEIKNYWNTRVKQRKRAGLPLYPPEIQQHISSRCHGQVQNAPWTANSPPTLKLLQPTNSSFTSAMPLLSENPYGSILQDPSRGKNISFQFPFLTSPAQNWPETFDLGPPPLSVKEELPSSQFSSNYGDEQMPHCNSGLLNDLLPESHGEEKLLSSTCLAIDLLGKLGYWDCPLGSSIVSTILLLLLLLLLPISGIKVIEEMLETDGRALIDMILTEMAMATPKTVVPDLYNNCSSGGSSGEFSNCPSSVTTDEEIELDMPQLEWSYVHGTGGF |
| >CgMYB70  FGGEMGRHSCCLKQKLRKGLWSPEEDEKLYNHIIRFGVGCWSSVPKLAGLFTLLTLRNLFFQSHGTNLTDLEFRLILCKFGAGLQRCGKSCRLRWINYLRPDLKRGSFSQEEEDLIIGLHEVLGNRWSQIAAQLPGRTDNEIKNFWNSCLKKKLRQRGIDPTTHKPLNEVETDQEKLNSDNSIHENLPIKPIFDPFPTLEFQAAAAAADQTGANLILYDQFHNSFISAPGFSEYGNIIDVSENNPFGNSSSISSNWNCNMRGDMNSLVCNEA |
| >CgMYB71  MVRAPCCEAMGLKKGPWTHEEDNILVSYILINGHGNWRALPKKAGLLRCGKSCRLRWTNYLRPDVKRGDFSKEEEETIIYMHAMLGNRHCDWIWSAIAAKLPGRTDNEIKNFWHTHLKKRLNPNQFIKMTKPRTANPDEPKEEDKFEDQISPVFIQNESYFSENITESNSVDMKQNSIDSFEEYFHDFDEILWSEEFTVTGEEEDGSIGQNFGQPSLSNEDSITVHSIYLEIGIMLLVAVFPPGSLGSGGCSVVSDLGLISEIDSYSTMRITSSKYADLIQKNNSSHTRKRKFNEFPDPPFV |
| >CgMYB72  MGRAPCCDKANVKKGPWSAEEDSKLKEFIEKYGTGGNWISLPHKAGLKRCGKSCRLRWLNYLRPNIKHGEFSDAEDRTICTLFASIGSRWSIIASQLPGRTDNDIKNHWNTKLKKKLLGISSYRKEKLTSSSTTAKPASSLFITITIFVFQPLHQH |
| >CgMYB73  YLCYTLLFPVNSATIYPSPASILSPNLSHILSSTLPFSRQNYSIEAFRRTTSSSQTRTPEDMGRSPCCDKVGLKKGPWTQEEDQKLLAYIEKHGHGSWRALPNKAGLQRCGKSCRLRWANYLRPDIKRGKFSMQEEQTIIQLHALLGNRWSAIATHLPRELIMR |
| >CgMYB74  MGRKPCCDKEGVKRGLWTIEEDQRLVDFILNNGIQCWRFVPKLAGLMRCGKSCRLRWINYLRPDLKRGALSEAEEEQIIQLHSNLGNRWSKIASHLPGRTDNEIKNIWNTRIKKKLKLQQSLDQITNNIEVERENHISKVEDKGLELEISTGSSKSSLFLELEQNWVQEKNIKQANVSISSSASASMDGNLNASSWEGESNLHGGSNQLLWADTTDYFSSWDAYNCVDDFLRYENYP |
| >CgMYB75  MEARKRDEFSKKRSWSSEEDKILLKYIESHGLRNWNIIGKNSGLSRDGKSCRLRWLNHLKPGLKKKLPFSKEEKFIIFSKHAALGNKWSKIAKQLPGRTDNEIKNFINTHMKKCLKTGTIPIYPREISMKYAQTGTYGLPKIASSDIADSVFQQWTQLVDLPSIQNTEEDGKDILLSSYLQNPQSSFESLTQPQLAAPQQALITGFSTVLSSEQCSNLSISALLYSDYSMNTDLYSLLSKFI |
| >CgMYB76  MGKQSCLHKQKLKKGLWSPEEDEKLLKHIAKYGHSSWSSVPKLAGLQRCGKSCRLRWINYLRPDLKRGAFLQEEEDLIIELHAALGNRSSQLHNWSFFVAFPSVTLFSPNRWSQIAAKLQGQTDNEIKNLWNSCIKKKLKQKGIDPNTHKPLSENEGTEDDKISSGTGNLKQTELDSALELMPGVEKDSTMNSIGHYSLSQLSCSTSTTIPSGSSNLLLWLKQSNREFGMNPELSCSSITSATPSVSSSNLYNSMDLKAIADLPYWEAGNPNSSSNKNCTSLNGDLCSWPDLTAERGNKYHLTGGNEEMKWSECLQSNLPVSSLPHCHNQPLHAGDLKVETHFDLESLNLCFQNH |
| >CgMYB77  MGRKPCCDKEGVKRGLWTIEEDQRLVDFILNNGIQCWRFVPKLAGLQINIFINPMLASLMRCGKSCRLRWINYLRPDLKRGALSEAEEEQIIQLHSNLGNRWSKIASHLPGRTDNEIKNIWNTRIKKKLKLQQSLDQITNNIEVERENHISKVEDKGLELEISTGSSKSSLFLELEQNWVQEKNIKQANVSISSSASASMDGNLNASSWEGESNLHGGSNQLLWADTTDYFSSWDAYNCVDDFLRYENYP |
| >CgMYB78  SSSSETNVQYDTIRTVKRKTRCETSGDICCCHRHHHRGSAITEEGTLDCSRRCNTKRYVEKYGEGNWNAVQKNLGLQRCGKSCRLRWANHLRPNLKKGAFSPEEEALIVQLHAQLGNKWARMALQLPGRTDNEIKNYWNTRIKRRQRVGLPLYPPEIQYQTALKNQKQGTFMLSQNQTQPKNFISHISPTTTTLFSNSSTRNNNANYLLGNFNLNPLPQPQPQPQSQPQQLPSPTFSTNMKLPSPQLFSSCAGDPKLPPLPTPSIMRQENNVLFDYLYQKPQGPE |
| >CgMYB79  MTGEPLKMEEKGPWTPQEDKLLTEHVKLHGEGRWNCVSRLTGLDGFNYLRPDLKRGKITPHEETIILELHAKWGNRWSTIARSLPGRTDNEIKNYWRTHFKKGKMTTNIERARAKFLKQQQEQKQQQQHQQLQLEVPQFEEIAITQLMQEIAYMCNMPYVFQGEEFTRKLMVSSDDRLGDGEAYYATWENLWNLDDLGLDMGDQALLFTTRFNVKIKKKHRGLRNAIV |
| >CgMYB80  MGRPPCCDKVGVKKGPWTPEEDIILVSYIQEHGPGNWKAIPTNSGLSRCSKSCRLRWTNYLRPGIKPIASYLPERTDNDIKNHWNTHMKKKLRRMENEGGMIAGSGFSKNQSIAKGQWEKRLQTDINMAKQALQEALSMEKPSFLYESKPSSSSHSSSTPPSSTTYASSTENISRLLENWMRKAPKNSAKSTQHSVKNFVNIGADSTSSDGTTTSVANNHIVSPEISQFQVDSKPFLDERFPLSLIETWLFDENNWGGNGSNNVLDFSLDDASHELF |
| >CgMYB81  MLNGRIGKQCRERWHNHLRPNIKKDTWSEEEDKILIQAHSEIGNKWAEIAKRLPGRTENSIKNHWNATKRRQFARRRSRSTKKPKSGASSLLQEYIKSLSLLNPSPASAPAAANTNDSAASTAEAAPPLPIALTSVSAWEDDFSDVDAELMMLDKKEMELMTDVCDVGYLFDQLGCGEGSSRSSSNMRDRLEGCLSMSCDEKVKKEKDLVEMINDHHNHNSSSSYSSACNEMLAA |
| >CgMYB82  MGRAPCCDKANVKKGPWSPEEDNKLKDYIENIDRIIFSEGRKFSLEIYMEIIVLKAVLGLLSGLKRCGKSCRLRWLNYLRPNIKHGNFSDDEDRIICSLFASIGSRWSIIASHLPGRTDNDIKNYWNTKLKKKLFGISPTQRKSSHQLKQQNQLLPPSLDHNILHPSTSTSLLSHHKYFPTIFSNTSTTSSPLVQVWPSQHYQTELNSSKLLVFGGDQLNCSFSEISFGRAMEFEPENYSLFNEIGDQKLLLGGISGGEIEAPLINSEFEEIKQVINPKNLYLDDPRRVTEASGNSFKPHERVMYYC |
| >CgMYB83  MGRPPCCDKAGIKKGPWTPEEDIILVSYIQEHGPGNWRLVPTNSGLMRCSKSCRLRWTNYLRPGIKRGNFSSHEEEIIIQLQALLGNRHCSSFSCQIHKLIGLLDNCPLTFQRTDNDIKNYWNTHLKKKIKKYQASVGSYITSSDSAGSTSHERVLGGYGADEIRNSCSSNSHTLTHSSSTYASSAENISRLLEGWMRSSPNQTLNDKKLQHQMKKESITFNSNSFIMNQLNFDVEVNSQLPLEGSVFAWDKTATDSVFEENKQFKMMTYDDNKLKTQSQQPA |
| >CgMYB84  MASLSALWLDFELFAWAGLFEKCCVGWLLYFHECEDYVRFLNMCSVEKDGDTKMPPEEQPDSSSNDERSWSGSPTSGEGIALKKGPWTSTEDAMLIDFVKKHGEGNWNAVQRNTGLPRCGKSCRLRWANHLRPDLKKGPLTREEEQFIIQMHAKIGNKWARMAALVSTHILS |
| >CgMYB85  MAMDAGAPFLPKAGLQRNGKSCRLRWINYLRPGLKRGIFTSEEEEIIMSLHAKLGNKWSRIAAHLPGRTDNEIKNYWNSYLKKKLMQLNHQHPMISSNELNESIHQTSKTENFQLESINQSSTSSCSIDNGSFKSSFPRVLFEEWLSPQDMGRDEFDENGLLEEMQSLDMFGEMIGDIDMNYDFIH |
| >CgMYB86  MGHHSCCNQQKVKRGLWSPEEDEKLIKYVTTHGYGCWSEVPDKAGLQRCGKSCRLRWAHIASHLPGRTDNEIKNYWNSWIKKKIRKPNTGTCSSSSPQCSSVQILPTLIPGFNSLNQFEPLANQTLTNSKPNDSNPIFPSSPIPLFMFDSECGGTVAKEDHELLLDVGNLNLVDVWNTNDNQNDHQVLSPLSTFVDQNYLPPLVDSMVAMVPPPGCIGEECEASGSQECFEKNELSEWVEAQQYPGFFIWDQTVQGQLGGDELPVSTPPTSNTDASVDSFPSTL |
| >CgMYB87  MVDESSNADKRIHINQEAEVRKGPWTMEEDLILINYIANHGEGVWNNLARSADKGNMNFGLVLREGLKRTGKSCRLRWLNYLRPDVRRGNITPEEQLLIMELHARWGNSNENRWEAVVENCEATPWKDDNEIKNYWRTRIQKKVKNGDAPDYSSQMIIDEASTSHTSSIEEGMALQSYPIHPNTNPEAYPPSCNSGELGENFWSMEDFWPIQSDQSLNGD |
| >CgMYB88  MVRAPCCERVGLNKGQWTPQEDKILITYIDLWAWKLESSSRKSWCGKSCRLRWTNYLNPGIKRGNFTKEEEDTIINLQQILGNRWSAIAAMLPGGTDNEIKNVWHTHLKKRLNPNQILQNSKRKIERRDNRSNSVIEFSSSSASESCSDFSSPRTESSSNVSERDNSNASRNDESTCDNGVEEIEESFWTEILSVNGCDSPLMEVDSSKNEDLSFWLRIFMEAETLQGFPEI |
| >CgMYB89  MASEATGLLCQRKLRCGKSCRLRWLNYLRPDIKHGSFTKEEDDMIFTLYNKIGSRWSVIASKLPGRTDNDVKNHWNTKLKKRVMEGRTTHSSETYHKSSTPLLPSQPLIPSAHSSSSTSCVLTPAIGNQEFQSSDDSLDASLQQEDHRGGSEYGLLWELDFGSITDLLSSSGSDDTLASIWTAHC |
| >CgMYB90  MSLISTNHLNSNCSTLEITTIDHPGLFSEVIAVLFELSYDMEFGKVWTYNGRAAFILNITKQSTGKPIFDPIRLRYVEEQVMSVVKAHNFLGELWRVDHSSLATVAMEDKRRDGHNNGGNGEVCEPYVSVESWKEMVYLVVNLLAFNPSSSPSWLLRPQFTFFFFVFFRSPFTFFRPQLIIAATHSLQINIDYAEVNIYRDSTYVMAHGCSYFEPDYVPLSEYIPPLEILDHHLILLARFYCRVCIDNETCDCTLVKVDGANRHGMLLKMVQTLSDLELVISKSNISSDNGWFMDVFYITDYYGQKLRDPTLISYIEHSLNVAQHQNGPNGSDQVKISHGNIVQVDHFASKCIALEMIFIDHPNIFSEVTTVLFNLSYAIDSGEMWTYNGRVACILYLKEQDGQPISDQARLDFLADYIARVVEAHHISSELWAVQLRGYYTCRTHTERRLHQLMQTCKDFEGGSPPLSFKDDQLSMTKNKEIKKWFGGADKGNKAVCRPQVSIDSWKERNYLVVNVRSSDRPKLMFDIVCVLTDLNFDVFHGSICSDGSETVQEYFVRLTNGSAVINEMEKECVARILAAAIDRRMVQMEVHIMDRHSLLLDVSRALRENSMSLKKVKFAKEDGLAVGTFYITDASPSTDANNIDQQRLETVCNEVGEACEAAWTPQEDQLLIEHVKLHGEGRWNSVSWLTANIVARIEEEWKSRLRWVNYLRPDLKRGKITAKEEAIIHELHARWGNRWSMIARSLPGRTDNEIKNYWRTHFKKSTKNVKCPSKILEATTGTKTTTSVMSKQYIKEETSSQTQMAIMPKEMEEMTFLCNNMPLMLHEGGFVGESTTSSEDRTSEENLLRHMGSLWNLDDMGDPTLTF |
| >CgMYB91  MKRKPFCDEDGLNKGAWTALEDKLLVSYINAKGEGKWTSVPTKAGLNRRGKSCRLRWSLIAGRLPGRTDNEIKNYWNTTLSKKLQNQNFAINMPNNKLLEPKLKPQENHASSSSMPALIRTKALRCTEASYPCFPLQIPASTDGVDQTKQISQQNFGDESMEKGKDDNLPNFDLVSFDDEMWFDGMEFEGLIRGDDDDDDGIMIEDNWTCGSLETDIWMNF |
| >CgMYB92  MMGDQEEPNSVDKMINNSNEEAEVVRKGPWTMEEDLILMNYIAIHGEGFWSNLARSAGLKRTGKSCRLRWLNYLRPDVRRGNITPEEQLLIMDLHSRWGNRIQKKVKDGETVNCSSQMMKYEGSSSHTGGLEEIIAQTSYPIYPDANPQEAYVPTYSESNPNETYLSAEDSGPYNPLMDYRAAP |
| >CgMYB93  PWTAEEDQILISYIHNHGHGNWRALPKLAGNYFFFFFFLLLLLSFVEDGNLKNRKKKSYIRFLFNVYLGLLRCKSCRLRWTNILDLILKEGTSPGKKRIQSLPYIRCLETKLFLPLWSAIAAKLPGRTENEIKNVWHTHLKKRLKKTDTETTQEPKRKTQIESNEETTTHS |
| >CgMYB94  GFVYLSICNSAAFRLLSLSKQSLCLTKLQEARRKMVRAPCCDKKGLKKGRWTVEEDEILMNYIAENGEGSWRSLPKNAGLLRCGKSCRWSVIAAQLPGRTDNEIKNYWNSHLSRRVHCFRRHGESKTFIYDIGSIPSPGKRRGGRTSRAAMMKNSSSSMVSGSIREDQISPNPNQNQVLNHTNNNLCISSSSSALSEKENSSLTFDFDFDLEEVLGPNGDIDAVSLEANEMGETRRATCSGEKEVELAMEEESGTMMTEMEKMLKWDFESLEAKLWEEDGSDMWPWLWDISGDIIFDEEPLGSWLLA |
| >CgMYB95  MGRAPCCDKAIVKKGPWSPEEDAKLKAYIEEHGTGNNWIALPQKIGLKRCGKSCRLRWLNYLRPNIKHGGFSEEEDRIICSLYLSFQEELITTSNYWNTRLKKKLLGRRRDLPPSHQLAPANQKLNDEGSNPNPNESSQNLTTSAIERLKLHIQLQGLHSPFSYGNASLWPNNKLLQTLNSTDHSIATAASPLKHFQQTNICNSMISNMQEHIVIPSSSCNLEAELHNLLDGYKENYQFTQVDCLKEIMNDIDQKGFDWWESNEFVEKLSSTSWDSALQPDTVFIQDDELGYDQ |
| >CgMYB96  MGRGRAPCCQKIGLHKGSWTPQEDIRLISYIRKHGHGNWRALPKQAGLPSLLSLKYLLLACCGAGRAAAFDGSTISVLTSSEAISPRKKKTPLSNSIDRSATNKYLGCFCTARWSKIASCLPGRTDNEIKNVWNTHLKKRLRHNNHPTQSPANMAARPEVDAPSSSSSGTAVTEGEELSFNKIEIPIEPQLDMWDILDSSLSPNLSPLPPPPPVEDDDILSDIPIDPDLWSDDTNIDSKTKEIDNSVGGKSADESKNWLAYLEEELGLFDEAETKETVELISMQVGESMGSYFHKRPSSSSTSLTALVDLTVVMHSPWSN |
| >CgMYB97  MFNQLDPRINRSPFTEEEEERLLASHRIHGNRWSVIARLFPGRTDNAVKNHWHVIMARKVRERSRFHGKRPCFSSSSLIMDDSSSNAQGKQGSLQEPQGFFSLVGNCYKKQQNCFLLGDFRSNSDEFQSWIHGFSGDFSLVGKKNPIEFYDFLQVNSDSNGTKCCSSVEDEKDEADQQEQSKARPFFDFLPVNGSI |
| >CgMYB98  MQEEQTIIQLHALLGNRWSAIATHLPKRTDNEIKNYWNTHLKKQLAKMGIDPVTHKPKSDALASADGHTRSTANLNHMAQWESARLEAEARLVRESKLRSSAPPSPFPPQYLPQPPPPVSMPTAASPSLDVLGAWQGEWPKPVVNSQAGSHNIDLESPTSTLSFSENMLPSRIPGMGTANDSTNWKCLKKPGFSLDTAEAFVNAEATSWLTGSCSGGFAAGFTGMLMGNTNKQNSTEGCDDSDIAGGSCVDVEEGEDEAEENKNYWNSIFNLVNSSSPSNSPPAVF |
| >CgMYB99  MQEEQTIIQLHALLGNRWSAIATHLPKRTDNEIKNYWNTHLKKQLAKMGIDPVTHKPKSDALASADGHTRSTANLNHMAQWESARLEAEARLVRESKLRSSAPPSPFPPQYLPQPPPPVSMPTAASPSLDVLGAWQGEWPKPVVNSQAGSHNIDLESPTSTLSFSENMLPSRIPGMGTANDSTNWKCLKKPGFSLDTAEAFVNAEATSWLTGSCSGGFAAGFTGMLMGNTNKQNSTEGCDDSDIAGGSCVDVEEGEDEAEENKNYWNSIFNLVNSSSPSNSPPAVF |
| >CgMYB100  MENLLVVMRMASRRGLGLLKRTRSWFITSRSMAMAVGELFLSLLDLNRCGKSCRLRWTNYLRPDIKRESSHLKRSRLSSISTLSLATKTFSSSPSRWSAIATHLPGRTDNEIKNYWNTHLKKKLIQMGFDPMTHQPRTDFSAALPHLLALANLVDHRPTSWLDSLKAEAAKLQFLQYFLQSANQPPQLELPLCYDYHSLLSNDNLNYEESKLPLMESPVSTLPPLIDASNPGDACSTSSCEGRGNSASATSFWPEFLLDDSFMNELA |
| >CgMYB101  MSLWPSSAVQCKSAIGHSSQRYIYKDKREREREMGRGRAPCCQKVGLNKGSWTLEEDQRLISYINRTAMKTGVHFLSSQAYCVVGRVVVFDGSTTFALTSSVVTSPRRKKTPSSTSMNCLEISKKWSKIAAHLPGRTDNEIKNVWNTHLKKRLNLKNFTPPSPTSMTKLHDDSLSPTSSSTPPFLFLMKLIAIALTKRHPS |
| >CgMYB102  MVPQHLSSTDHGLGNRYIEAQAQEEANSPSPSSSKSFLSHMASLPIKPSCSFSTHHLFHFMGGSDTNEVLEEDNNRADAEEANVNDCTGQSNLCVRGHWRPEEDCKLKALSCRFRRFNQLDPTISRSAFTEEKEKLMAAHRHYGNKWAMIARLFPGRIDNAVKNHWHVIMARKYRKQSTAYRRRKLNQAMDQRSEEIIPKPLLIMMEKNSSLIHAGSYIQEKNPLISFL |
| >CgMYB103  MGRGRAFCCGVMEAKKGWWRAEEDSKLVSYIQKYGHQNWGTSRVAAVWQELPAEAGQLPPPRQARQLHPIGGRNHNEAPQRARKPVNSLHYSIYIFPKQNFRVRHNPFGHFSSSWSKIASLLPGRTDNKIKNIWNTYLRKRTARRMHRDAGTNDCVISSNILPSKPSELATAAENNDLIVPFERRHIWALGGCR |
| >CgMYB104  EHGPKRKTSASLLTSRRTERGAGALPKAAGLLRCGKSCRLRWINYLRPDLKRGNFTEEEDELIIKLHSLLGNKSMVFDSGEAAGEDRQREKNYWNTHIRRKLMSSGIDPATHRPLHEQQAKTTISFIKGEEKVEEFEGREEGKKSSSNSSEEMSAWLRRRPEEENKQWRCPDLNLELCMSPPPSLKQQALCQEPVKSEELSLCFNCRLGVNKSSECKCGNGFLGLRSGVLDYRRLGPTES |

**Supplementary Table 2|** *CgMYB* gene sequences were used in RT-qPCR;

| **DNA sequences** |
| --- |
| >CgMYB91  ATGAAGAGGAAGCCATTCTGTGACGAGGATGGGCTAAACAAGGGAGCTTGGACGGCTCTGGAAGACAAGCTGTTGGTCTCTTATATAAATGCCAAAGGCGAAGGCAAGTGGACGAGCGTTCCAACGAAAGCAGGGCTGAACAGACGTGGAAAGAGCTGCAGGCTCAGGTGGTCTTTAATTGCTGGAAGGTTACCTGGTCGAACAGATAATGAGATCAAGAACTATTGGAATACAACTTTATCAAAAAAGTTGCAAAATCAAAACTTTGCAATCAACATGCCAAATAATAAGCTATTGGAGCCAAAATTAAAACCTCAAGAAAATCATGCTTCTTCTTCTTCAATGCCTGCTTTGATTCGAACCAAGGCATTGCGGTGCACAGAAGCTTCTTATCCTTGCTTTCCATTACAGATTCCAGCTTCTACAGATGGAGTTGATCAAACCAAACAAATAAGTCAGCAAAACTTTGGAGATGAATCAATGGAGAAAGGGAAGGATGATAATTTGCCAAACTTTGATTTGGTTTCTTTTGATGATGAAATGTGGTTTGATGGAATGGAATTTGAGGGCTTAATAAGGGGTGATGATGATGATGATGATGGAATTATGATTGAAGATAATTGGACTTGTGGCTCATTGGAGACAGATATTTGGATGAATTTT |
| >CgMYB32  GCCTTTGAGAGGAGGAAATTCACAAATTTAGGGAGGGGGACAGAGATGGGGAGGTCTCCATGTTGCGAGAAAGCGCACACCAACAAAGGGGCATGGACGAAAGAGGAGGATGAGAGGCTCATCGCGCACATCCGCGCTCACGGCGAAGGATCCTGGCGCTCGCTCCCCAAAGCTGCCGGCCTTCTTCGCTGCGGCAAAAGCTGCCGCCTTCGTTGGATCAACTATCTTCGCCCTGACCTCAAGCGCGGCAACTTCACCGATGATGAGGACGAGCTCATCATCAAGCTTCACAGTCTCCTGGGCAACAAATGGTCTTTGATAGCTGGAAGGCTACCGGGAAGAACTGATAACGAGATCAAGAACTACTGGAACACTCATATACGGCGGAAGCTGCTCAATAGGGGAATAGATCCGGCGACGCACCGTCCCATTTCCGCCGCCTCCACCTCAAATCTCTCCATAACAGATAAGAGAAACGGAGTCAATAGAGTATCTGCTCTGCTGCCGCGCTGTCCTGATCTGAATCTCGACCTCTGCATCAGCCCTCCATTGGAGGAGCAGTTGCAGCAAGATTTGTTGGAGGAGAGTAACTGCGGAGGCGAGTTTCTTAGGCTTGGCAGCAGCTTTCTGCTTGATTACAGAAGCTTGGAGATGAAGTGA |

**Supplementary Table 3|** RT-qPCR primers.

| Gene |  | Primer**（5'to3'）** |
| --- | --- | --- |
| CgMYB91 | F | CCTTGCTTTCCATTACAGATTCCA |
|  | R | TCCATTCCATCAAACCACATTTCA |
| CgMYB32 | F | CCTCCACCTCAAATCTCTCCATA |
|  | R | CCGCAGTTACTCTCCTCCAA |
| Actin | F | ATTCAGCCTCTAGTTTGCGATAA |
|  | R | CAGCAAATCCAGCCTAACAAATG |

The RT-qPCR primer of *CgMYB* were designed by the Primer Premier5 software.

F, forward; R, reverse. The primers were synthesized by Sangon Biotech (Fuzhou,China)

**Supplementary Table 4|** Renaming and protein characterization prediction of *CgMYB* genes in *C.goeringii*

| **Gene ID** | **Name** | **AA** | **Mv** | **PI** | **II** | **AI** | **GRAVY** | **Locations** |
| --- | --- | --- | --- | --- | --- | --- | --- | --- |
| GL13098 | CgMYB1 | 289 | 31.99 | 6.05 | 47.45 | 76.06 | -0.444 | Nucleus. |
| GL01705 | CgMYB2 | 258 | 30.10 | 8.39 | 65.39 | 61.98 | -0.88 | Nucleus. |
| GL09556 | CgMYB3 | 301 | 33.78 | 7.67 | 49.56 | 66.78 | -0.715 | Nucleus. |
| GL33040 | CgMYB4 | 290 | 32.03 | 6.4 | 58.9 | 66.34 | -0.684 | Nucleus. |
| GL15493 | CgMYB5 | 288 | 33.24 | 7.09 | 59.89 | 66.32 | -0.753 | Nucleus. |
| GL11185 | CgMYB6 | 337 | 38.27 | 5.65 | 49.49 | 77.54 | -0.616 | Nucleus. |
| GL15959 | CgMYB7 | 242 | 28.44 | 7.32 | 66.95 | 61.24 | -0.924 | Cytoplasm. Nucleus. |
| GL20991 | CgMYB8 | 230 | 26.57 | 6.46 | 57.57 | 66.52 | -0.722 | Nucleus. |
| GL00356 | CgMYB9 | 295 | 33.70 | 8.63 | 60.95 | 70.17 | -0.653 | Nucleus. |
| GL09143 | CgMYB10 | 324 | 36.78 | 6.4 | 44.14 | 86.08 | -0.587 | Nucleus. |
| GL14957 | CgMYB11 | 249 | 28.30 | 8.91 | 47.38 | 97.15 | -0.453 | Nucleus. |
| GL12694 | CgMYB12 | 363 | 41.70 | 9.34 | 55.33 | 81.43 | -0.478 | Nucleus. |
| GL18687 | CgMYB13 | 244 | 28.00 | 9.12 | 55.97 | 65.98 | -0.693 | Nucleus. |
| GL26789 | CgMYB14 | 357 | 39.02 | 6.52 | 58.12 | 61.85 | -0.642 | Nucleus. |
| GL26794 | CgMYB15 | 357 | 39.02 | 6.52 | 58.12 | 61.85 | -0.642 | Nucleus. |
| GL01909 | CgMYB16 | 303 | 33.39 | 8.6 | 56.56 | 77.29 | -0.485 | Nucleus. |
| GL13049 | CgMYB17 | 217 | 24.15 | 8.72 | 55.22 | 75.67 | -0.566 | Nucleus. |
| GL21544 | CgMYB18 | 747 | 83.21 | 8.94 | 59.51 | 72.9 | -0.378 | Nucleus. |
| GL08371 | CgMYB19 | 314 | 35.40 | 8.14 | 59.24 | 71.43 | -0.704 | Nucleus. |
| GL18907 | CgMYB20 | 327 | 36.27 | 6.90 | 51.80 | 83.58 | -0.572 | Nucleus. |
| GL19745 | CgMYB21 | 233 | 25.80 | 8.47 | 55.93 | 68.76 | -0.544 | Nucleus. |
| GL32762 | CgMYB22 | 242 | 25.81 | 9.09 | 66.34 | 68.64 | -0.51 | Nucleus. |
| GL02225 | CgMYB23 | 210 | 23.97 | 7.06 | 58.37 | 68.33 | -0.67 | Nucleus. |
| GL17640 | CgMYB24 | 225 | 26.28 | 9.32 | 58.55 | 62 | -0.911 | Nucleus. |
| GL14479 | CgMYB25 | 218 | 24.96 | 8.45 | 62.79 | 72.52 | -0.917 | Nucleus. |
| GL00279 | CgMYB26 | 191 | 21.59 | 9.45 | 47.71 | 85.34 | -0.563 | Nucleus. |
| GL11454 | CgMYB27 | 175 | 19.97 | 10.17 | 48.17 | 80.46 | -0.642 | Nucleus. |
| GL15370 | CgMYB28 | 335 | 37.09 | 6.67 | 52.46 | 82.45 | -0.591 | Nucleus. |
| GL03388 | CgMYB29 | 333 | 37.74 | 9.25 | 55.27 | 61.53 | -0.799 | Cytoplasm. Nucleus. |
| GL17601 | CgMYB30 | 264 | 30.31 | 8.89 | 76.03 | 68.03 | -0.91 | Nucleus. |
| GL08364 | CgMYB31 | 242 | 28.15 | 5.95 | 65.37 | 58.51 | -1.002 | Nucleus. |
| GL18847 | CgMYB32 | 219 | 24.93 | 9.26 | 49.14 | 84.7 | -0.661 | Nucleus. |
| GL10789 | CgMYB33 | 314 | 34.81 | 7.05 | 57.17 | 71.15 | -0.65 | Nucleus. |
| GL03281 | CgMYB34 | 251 | 28.12 | 8.51 | 52.35 | 78.92 | -0.468 | Nucleus. |
| GL04077 | CgMYB35 | 224 | 25.92 | 9.91 | 62.28 | 61.38 | -0.834 | Nucleus. |
| GL17502 | CgMYB36 | 273 | 31.16 | 9.67 | 51.28 | 76.81 | -0.674 | Nucleus. |
| GL07948 | CgMYB37 | 423 | 47.47 | 7.38 | 63.11 | 62.53 | -0.642 | Cytoplasm. Nucleus. |
| GL18686 | CgMYB38 | 281 | 32.45 | 10.87 | 67.65 | 75.3 | -0.691 | Nucleus. |
| GL04647 | CgMYB39 | 259 | 29.79 | 6.35 | 58.3 | 68.07 | -0.722 | Nucleus. |
| GL12678 | CgMYB40 | 560 | 64.54 | 5.51 | 50.25 | 89.12 | -0.388 | Nucleus. |
| GL33573 | CgMYB41 | 344 | 38.27 | 9.83 | 59.58 | 65.06 | -0.611 | Nucleus. |
| GL33574 | CgMYB42 | 344 | 38.27 | 9.83 | 59.58 | 65.06 | -0.611 | Nucleus. |
| GL18798 | CgMYB43 | 256 | 28.84 | 8.74 | 60.18 | 61.76 | -0.744 | Nucleus. |
| GL24654 | CgMYB44 | 357 | 40.11 | 6.47 | 52.78 | 58.54 | -0.724 | Nucleus. |
| GL08281 | CgMYB45 | 253 | 28.90 | 5.71 | 72.57 | 67.87 | -0.682 | Nucleus. |
| GL07683 | CgMYB46 | 308 | 34.79 | 5.97 | 53.12 | 57.31 | -0.786 | Nucleus. |
| GL09059 | CgMYB47 | 543 | 59.80 | 6.11 | 58.03 | 67.94 | -0.511 | Cytoplasm. Nucleus. |
| GL16107 | CgMYB48 | 335 | 36.97 | 5.82 | 44 | 66.69 | -0.627 | Nucleus. |
| GL13772 | CgMYB49 | 332 | 36.95 | 6.86 | 63.39 | 77.02 | -0.655 | Nucleus. |
| GL01165 | CgMYB50 | 427 | 47.76 | 7.64 | 67.53 | 68.5 | -0.621 | Nucleus. |
| GL06535 | CgMYB51 | 224 | 26.65 | 8.87 | 61.68 | 62.72 | -0.915 | Nucleus. |
| GL03580 | CgMYB52 | 296 | 32.35 | 9.84 | 58.95 | 66.15 | -0.591 | Nucleus. |
| GL20296 | CgMYB53 | 258 | 29.74 | 8.94 | 52.83 | 76.36 | -0.831 | Nucleus. |
| GL11270 | CgMYB54 | 323 | 36.56 | 6.79 | 62.31 | 69.47 | -0.753 | Nucleus. |
| GL13967 | CgMYB55 | 232 | 26.14 | 6.95 | 56.31 | 73.23 | -0.581 | Nucleus. |
| GL32419 | CgMYB56 | 323 | 36.92 | 9.06 | 59.97 | 70.37 | -0.741 | Cytoplasm. Nucleus. |
| GL12169 | CgMYB57 | 324 | 36.70 | 4.76 | 53.95 | 75.19 | -0.806 | Nucleus. |
| GL14407 | CgMYB58 | 321 | 36.14 | 8.75 | 47.39 | 67.48 | -0.659 | Nucleus. |
| GL15248 | CgMYB59 | 285 | 31.73 | 8.77 | 58.31 | 72.91 | -0.578 | Nucleus. |
| GL17209 | CgMYB60 | 270 | 30.86 | 8.71 | 54.67 | 69.7 | -0.913 | Nucleus. |
| GL18520 | CgMYB61 | 290 | 31.95 | 5.86 | 48.62 | 83.17 | -0.373 | Nucleus. |
| GL00724 | CgMYB62 | 283 | 31.13 | 5.88 | 58.15 | 63.82 | -0.525 | Nucleus. |
| GL03887 | CgMYB63 | 262 | 29.53 | 5.92 | 55.94 | 81.56 | -0.439 | Nucleus. |
| GL08701 | CgMYB64 | 251 | 28.51 | 6.33 | 42.14 | 82.79 | -0.615 | Nucleus. |
| GL10704 | CgMYB65 | 356 | 40.30 | 7.33 | 61.03 | 75.39 | -0.638 | Nucleus. |
| GL10682 | CgMYB66 | 222 | 26.13 | 8.77 | 59.75 | 62.84 | -0.895 | Nucleus. |
| GL17323 | CgMYB67 | 375 | 42.31 | 6.03 | 54.98 | 61.92 | -0.73 | Nucleus. |
| GL12688 | CgMYB68 | 687 | 78.10 | 8.64 | 62.19 | 84.32 | -0.442 | Nucleus. |
| GL16226 | CgMYB69 | 392 | 43.43 | 6.76 | 54.3 | 80.89 | -0.407 | Cytoplasm. |
| GL17053 | CgMYB70 | 272 | 30.78 | 6.72 | 49.61 | 80 | -0.451 | Nucleus. |
| GL01696 | CgMYB71 | 302 | 34.55 | 5.36 | 52.43 | 71.99 | -0.578 | Nucleus. |
| GL13452 | CgMYB72 | 156 | 17.64 | 9.84 | 42.69 | 74.49 | -0.564 | Nucleus. |
| GL07937 | CgMYB73 | 164 | 18.73 | 9.78 | 69.48 | 78.6 | -0.52 | Nucleus. |
| GL16686 | CgMYB74 | 237 | 27.22 | 6.14 | 44.49 | 81.48 | -0.698 | Nucleus. |
| GL10755 | CgMYB75 | 242 | 27.52 | 9.42 | 48.91 | 83.88 | -0.523 | Cytoplasm. Nucleus. |
| GL17768 | CgMYB76 | 355 | 39.57 | 6.89 | 64.01 | 74.76 | -0.627 | Nucleus. |
| GL03052 | CgMYB77 | 250 | 28.68 | 6.14 | 45.34 | 85.44 | -0.603 | Nucleus. |
| GL10966 | CgMYB78 | 285 | 32.53 | 9.74 | 62.39 | 61.61 | -0.928 | Cytoplasm. Nucleus. |
| GL09098 | CgMYB79 | 228 | 26.86 | 8.74 | 47.67 | 73.16 | -0.826 | Cytoplasm. Nucleus. |
| GL01504 | CgMYB80 | 277 | 30.96 | 8.58 | 45.78 | 61.99 | -0.752 | Cytoplasm. Nucleus. |
| GL17190 | CgMYB81 | 235 | 26.24 | 6.66 | 64.33 | 66.55 | -0.763 | Nucleus. |
| GL19914 | CgMYB82 | 307 | 35.02 | 8.56 | 52.41 | 83.52 | -0.491 | Nucleus. |
| GL03659 | CgMYB83 | 283 | 32.05 | 8.82 | 46.92 | 66.86 | -0.704 | Nucleus. |
| GL14152 | CgMYB84 | 172 | 19.55 | 6.21 | 48.66 | 69.24 | -0.476 | Nucleus. |
| GL21042 | CgMYB85 | 186 | 21.45 | 5.52 | 53.77 | 70.32 | -0.656 | Nucleus. |
| GL08333 | CgMYB86 | 284 | 31.49 | 4.84 | 47.21 | 68.59 | -0.534 | Nucleus. |
| GL08840 | CgMYB87 | 220 | 25.32 | 4.98 | 51.57 | 67.41 | -0.89 | Nucleus. |
| GL12038 | CgMYB88 | 232 | 26.58 | 5.2 | 72.85 | 68.92 | -0.764 | Nucleus. |
| GL02838 | CgMYB89 | 185 | 20.61 | 6.65 | 53.58 | 71.73 | -0.599 | Cytoplasm. Nucleus. |
| GL04596 | CgMYB90 | 869 | 99.48 | 5.71 | 46.23 | 84.44 | -0.277 | Nucleus. |
| GL19121 | CgMYB91 | 221 | 25.08 | 4.92 | 41.34 | 64.93 | -0.76 | Nucleus. |
| GL19940 | CgMYB92 | 184 | 20.93 | 4.96 | 53.56 | 65.22 | -0.802 | Nucleus. |
| GL00835 | CgMYB93 | 171 | 20.19 | 9.84 | 46.44 | 90.7 | -0.485 | Nucleus. |
| GL08542 | CgMYB94 | 307 | 34.62 | 5.29 | 57.28 | 70.88 | -0.565 | Nucleus. |
| GL04782 | CgMYB95 | 294 | 33.57 | 5.87 | 53.48 | 77.35 | -0.697 | Cytoplasm. Nucleus. |
| GL14956 | CgMYB96 | 320 | 35.05 | 5.66 | 60.17 | 78.69 | -0.483 | Nucleus. |
| GL13890 | CgMYB97 | 196 | 22.49 | 6.59 | 62.52 | 56.68 | -0.767 | Cytoplasm. Nucleus. |
| GL26791 | CgMYB98 | 286 | 30.89 | 5.17 | 58.97 | 62.87 | -0.57 | Nucleus. |
| GL26792 | CgMYB99 | 286 | 30.89 | 5.17 | 58.97 | 62.87 | -0.57 | Nucleus. |
| GL16961 | CgMYB100 | 267 | 30.15 | 8.98 | 56.62 | 84.12 | -0.33 | Nucleus. |
| GL01433 | CgMYB101 | 201 | 22.56 | 10.17 | 43.78 | 71.29 | -0.49 | Nucleus. |
| GL00776 | CgMYB102 | 229 | 26.25 | 8.97 | 58.31 | 66.55 | -0.717 | Nucleus. |
| GL16742 | CgMYB103 | 194 | 22.19 | 10.42 | 58.48 | 66.96 | -0.741 | Nucleus. |
| GL10239 | CgMYB104 | 240 | 27.25 | 8.95 | 79.35 | 65.88 | -0.905 | Cytoplasm. Nucleus. |

AA (Amino acids), Molecular weight (Mw), theoretical isoelectric point values(pI), grand average of hydrophilic (GRAVY), aliphatic index(AI) and instability index(II).

**Supplementary Table 5|** Conserved motifs in CgMYB proteins via MEME website.

| Motif | Sequences | E-value | Sites | Width |
| --- | --- | --- | --- | --- |
| 1 | AGLLRCGKSCRLRWTNYLRPDJKRGNFTE | 3.0e-1919 | 93 | 29 |
| 2 | WSLIARHLPGRTDNEIKNYWNTHLKK | 4.5e-1918 | 95 | 26 |
| 3 | KKGPWTPEEDEKLVSYIQKHG | 1.9e-1092 | 95 | 21 |
| 4 | EEEJIIQLHALLGNR | 3.0e-626 | 93 | 15 |
| 5 | MGRAPCCDKAG | 3.2e-374 | 59 | 11 |
| 6 | KLLQMGIDPVTHRPLSDALA | 6.4e-269 | 33 | 20 |
| 7 | ANLNHMAQWESARLEAEARLVRESKLRSSAPPSPFPPQYLPQPPPPVSMP | 5.4e-194 | 6 | 50 |
| 8 | STLSFSENMLPSRIPGMGTANDSTNWKCLKKPGFSLDTAEAFVNAEATSW | 7.2e-194 | 6 | 50 |
| 9 | HGNWRSLP | 1.8e-183 | 77 | 8 |
| 10 | VLGAWQGEWPKPVVNSQAGSHNIDLESPT | 4.3e-109 | 6 | 29 |

Red “w” represents the conserved tryptophan (Trp, w) residues

**Supplementary Table 6|** *Ka/Ks* analysis of *CgMYB* genes

| **Sequence 1** | **Sequence 2** | ***K*a** | ***K*s** | ***K*a/*K*s** |
| --- | --- | --- | --- | --- |
| CgMYB55 | CgMYB89 | 0.311663 | 0.793955 | 0.392545 |
| CgMYB87 | CgMYB92 | 0.23278 | 1.14167 | 0.203895 |
| CgMYB58 | CgMYB56 | 0.439453 | 3.247601 | 0.135316 |
| CgMYB2 | CgMYB39 | 0.40829 | NaN | NaN |
| CgMYB30 | CgMYB27 | 0.203931 | 3.555259 | 0.05736 |
| CgMYB67 | CgMYB36 | 0.292689 | 1.127883 | 0.259503 |
| CgMYB67 | CgMYB9 | 0.216139 | 0.82459 | 0.262117 |
| CgMYB14 | CgMYB73 | 0.082079 | 0.934979 | 0.087787 |
| CgMYB44 | CgMYB37 | 0.231357 | 0.762912 | 0.303256 |
| CgMYB101 | CgMYB96 | 0.309231 | 0.996076 | 0.310449 |
| CgMYB39 | CgMYB5 | 0.349629 | 1.396978 | 0.250275 |
| CgMYB10 | CgMYB11 | 0.349963 | 1.582752 | 0.22111 |
| CgMYB71 | CgMYB31 | 0.264854 | 2.178013 | 0.121604 |
| CgMYB10 | CgMYB28 | 0.268329 | 1.024752 | 0.261848 |
| CgMYB28 | CgMYB20 | 0.023105 | 0.026207 | 0.881627 |
| CgMYB56 | CgMYB59 | 0.267006 | 1.518154 | 0.175875 |
| CgMYB83 | CgMYB63 | 0.402914 | 2.85196 | 0.141276 |
| CgMYB6 | CgMYB68 | 0.301976 | 1.751804 | 0.17238 |
| CgMYB69 | CgMYB78 | 0.612753 | NaN | NaN |
| CgMYB95 | CgMYB65 | 0.234452 | 1.07874 | 0.217339 |
| CgMYB37 | CgMYB36 | 0.448699 | 3.444101 | 0.130281 |
| CgMYB36 | CgMYB35 | 0.354902 | 1.873674 | 0.189415 |

**Supplementary Table 7|** The FPKM values of *CgMYB* genes in different flower tissues.

| ID | Y-se | Y-pe | Y-lip | Y-col | R-se | R-pe | R-lip | R-col |
| --- | --- | --- | --- | --- | --- | --- | --- | --- |
| CgMYB1 | 0.14 | 0 | 0.07 | 0 | 2.78 | 2.19 | 0.94 | 2.08 |
| CgMYB2 | 0.41 | 0.4 | 0.18 | 1.19 | 1.01 | 0.22 | 0.63 | 1.49 |
| CgMYB3 | 2.52 | 1.08 | 2.37 | 1 | 0.28 | 0.32 | 1.74 | 0 |
| CgMYB4 | 4.45 | 5.01 | 3.03 | 10.95 | 6.48 | 5.48 | 3.98 | 79.21 |
| CgMYB5 | 0.12 | 0.39 | 0.97 | 0.9 | 0 | 0.11 | 0.36 | 0.29 |
| CgMYB6 | 0.12 | 0 | 1.75 | 0.12 | 0 | 0.05 | 4.12 | 0.61 |
| CgMYB7 | 0.26 | 0.32 | 0.51 | 0.42 | 0 | 0.07 | 0.14 | 4.14 |
| CgMYB8 | 1.29 | 1.28 | 0.18 | 2.77 | 0.68 | 0.43 | 0.36 | 0.56 |
| CgMYB9 | 0.07 | 0.13 | 0 | 0.41 | 0 | 0 | 0 | 11.07 |
| CgMYB10 | 0 | 0 | 0 | 0 | 0 | 0 | 0 | 0 |
| CgMYB11 | 0 | 0 | 0.08 | 0 | 0.07 | 0 | 0 | 0.32 |
| CgMYB12 | 0 | 0 | 0 | 0 | 0 | 0 | 0 | 0.06 |
| CgMYB13 | 0 | 0 | 0 | 0 | 0 | 0 | 0 | 0.09 |
| CgMYB14 | 293.31 | 264.02 | 293.95 | 232.32 | 1.36 | 9.35 | 14.26 | 23.2 |
| CgMYB15 | 293.31 | 264.02 | 293.95 | 232.32 | 1.36 | 9.35 | 14.26 | 23.2 |
| CgMYB16 | 33.74 | 18.07 | 29.18 | 24.85 | 183.84 | 236.97 | 238.73 | 110.97 |
| CgMYB17 | 0 | 0 | 0.48 | 0 | 0 | 0 | 0.15 | 0.5 |
| CgMYB18 | 18.52 | 19.21 | 19.6 | 17.96 | 16.17 | 14.52 | 14.75 | 25.18 |
| CgMYB19 | 55.21 | 107.58 | 42.67 | 57.62 | 15.31 | 42.78 | 119.21 | 29.55 |
| CgMYB20 | 0 | 0 | 0 | 0 | 0 | 0 | 0 | 0 |
| CgMYB21 | 2.81 | 1.35 | 0 | 0 | 0.22 | 0 | 0 | 0.09 |
| CgMYB22 | 0 | 0 | 0.08 | 1.1 | 0.14 | 0.13 | 0 | 23.08 |
| CgMYB23 | 0.92 | 0.6 | 0.91 | 4.81 | 2.49 | 1.38 | 0.78 | 15.36 |
| CgMYB24 | 0.09 | 0 | 0 | 0 | 0 | 0 | 0 | 0 |
| CgMYB25 | 43.62 | 108.43 | 134.01 | 210.15 | 22.26 | 27.73 | 110.37 | 99.09 |
| CgMYB26 | 0 | 0 | 0.11 | 0 | 0 | 0 | 0 | 0 |
| CgMYB27 | 29.81 | 49.28 | 44.54 | 30.27 | 51.76 | 42.66 | 39.1 | 41.95 |
| CgMYB28 | 0 | 0 | 0 | 0 | 0 | 0 | 0 | 0 |
| CgMYB29 | 11.61 | 11.44 | 11.42 | 0.29 | 0.38 | 0.07 | 0.46 | 0.3 |
| CgMYB30 | 12.09 | 34.58 | 30.6 | 15.35 | 16.83 | 20.86 | 14.12 | 21.02 |
| CgMYB31 | 0 | 0 | 0 | 0 | 0 | 0 | 0 | 0 |
| CgMYB32 | 27.33 | 18.55 | 15.45 | 24.2 | 64.85 | 92.94 | 97.5 | 91.85 |
| CgMYB33 | 7.57 | 7.18 | 6.49 | 2.46 | 1.91 | 1.71 | 1.77 | 0.53 |
| CgMYB34 | 0 | 0 | 0 | 0 | 0 | 0.26 | 0 | 0 |
| CgMYB35 | 0 | 0 | 0 | 0 | 0 | 0 | 0 | 0.96 |
| CgMYB36 | 0 | 0 | 0 | 0 | 0 | 0 | 0 | 0.08 |
| CgMYB37 | 0.05 | 0.06 | 0.41 | 0.06 | 0 | 0 | 0.88 | 0.03 |
| CgMYB38 | 0 | 0 | 0 | 0 | 0 | 0 | 0 | 0 |
| CgMYB39 | 0.24 | 0 | 0 | 0 | 0 | 0 | 0 | 0.16 |
| CgMYB40 | 0.56 | 0.32 | 0.24 | 0.1 | 0.24 | 0.05 | 0.03 | 0.11 |
| CgMYB41 | 58.91 | 49.15 | 55.51 | 37.92 | 0.45 | 0 | 1.88 | 2.36 |
| CgMYB42 | 58.91 | 49.15 | 55.51 | 37.92 | 0.45 | 0 | 1.88 | 2.36 |
| CgMYB43 | 0 | 0.08 | 0.29 | 26.3 | 0 | 0.04 | 0.06 | 6.21 |
| CgMYB44 | 0 | 0.1 | 0.11 | 0.22 | 200.95 | 166.95 | 64.48 | 3.9 |
| CgMYB45 | 0 | 0.08 | 0.08 | 0.24 | 1.01 | 1.66 | 0.39 | 0.42 |
| CgMYB46 | 0 | 0 | 0 | 0 | 0 | 0 | 0 | 0 |
| CgMYB47 | 9.83 | 5.71 | 8.14 | 13.02 | 1.97 | 1.25 | 2.8 | 56.82 |
| CgMYB48 | 0.48 | 0.34 | 1.17 | 0.65 | 0.74 | 0.93 | 2.02 | 2.7 |
| CgMYB49 | 4.7 | 1.61 | 7.11 | 4.77 | 261.78 | 95.88 | 84.32 | 13.59 |
| CgMYB50 | 0.48 | 1.77 | 8.08 | 17.01 | 2.77 | 4.05 | 13.1 | 35.19 |
| CgMYB51 | 0.09 | 0.44 | 3.68 | 0.9 | 0.39 | 1.75 | 0 | 0.26 |
| CgMYB52 | 44.73 | 25.31 | 16.76 | 8.82 | 8.46 | 9.18 | 13.24 | 12.57 |
| CgMYB53 | 0 | 0 | 0 | 0 | 0.46 | 0 | 0 | 0.16 |
| CgMYB54 | 14.48 | 7.51 | 35.37 | 15.27 | 3.3 | 5.12 | 17.56 | 11.46 |
| CgMYB55 | 1.91 | 1.27 | 3.02 | 4.16 | 3.2 | 3.39 | 8.63 | 3.24 |
| CgMYB56 | 0.06 | 0.12 | 0.18 | 0.18 | 0 | 0 | 0 | 0 |
| CgMYB57 | 10.07 | 2.74 | 5.72 | 6.88 | 0 | 0 | 1.03 | 3.94 |
| CgMYB58 | 0 | 0 | 0 | 0.06 | 0 | 0 | 0 | 0.19 |
| CgMYB59 | 0 | 0 | 0 | 0 | 0 | 0 | 0 | 0 |
| CgMYB60 | 0 | 0 | 0 | 0 | 11.52 | 6.23 | 7.73 | 5.68 |
| CgMYB61 | 0.07 | 0.07 | 0.14 | 0.07 | 0 | 0 | 0 | 0.07 |
| CgMYB62 | 0 | 0 | 0.07 | 0.07 | 0 | 0 | 0.06 | 0.22 |
| CgMYB63 | 0 | 0 | 0.04 | 0 | 0 | 0 | 0 | 0.08 |
| CgMYB64 | 0.5 | 0.93 | 0.89 | 0.32 | 1.09 | 0.58 | 1.11 | 0 |
| CgMYB65 | 0 | 0 | 0 | 0.06 | 0 | 0 | 0 | 0.12 |
| CgMYB66 | 41.74 | 28.55 | 122.28 | 27.5 | 24.18 | 46.41 | 90.08 | 43.07 |
| CgMYB67 | 1.03 | 0.62 | 9.04 | 0.59 | 0.93 | 2.88 | 13.7 | 12.05 |
| CgMYB68 | 0.24 | 0.18 | 0.35 | 0.25 | 5.4 | 2.04 | 0.79 | 0.99 |
| CgMYB69 | 13.22 | 15.22 | 18.78 | 17.81 | 31.12 | 26.71 | 35.07 | 25.43 |
| CgMYB70 | 1.51 | 0.45 | 0.72 | 0.86 | 0.4 | 0.06 | 0.12 | 0.19 |
| CgMYB71 | 1.77 | 1.88 | 2.39 | 2.85 | 0.25 | 0.11 | 0.28 | 0.52 |
| CgMYB72 | 0 | 0 | 0 | 0 | 0 | 0 | 0.57 | 0.3 |
| CgMYB73 | 13.86 | 6.24 | 7.5 | 0.66 | 0.78 | 1.06 | 9.17 | 2.78 |
| CgMYB74 | 0 | 0 | 0 | 0.17 | 12.41 | 4.04 | 0.17 | 0.61 |
| CgMYB75 | 0.26 | 0 | 0 | 0 | 0 | 0 | 0 | 0.18 |
| CgMYB76 | 5.93 | 3.77 | 6.69 | 5.81 | 1813.89 | 586.68 | 241.8 | 25.11 |
| CgMYB77 | 0 | 0 | 0 | 0 | 11.8 | 5.39 | 0.51 | 0.51 |
| CgMYB78 | 0 | 0 | 0 | 0 | 0 | 0 | 0 | 22.08 |
| CgMYB79 | 0 | 0.26 | 0.38 | 0.11 | 0 | 1.02 | 0.07 | 0.13 |
| CgMYB80 | 0 | 0.05 | 1.76 | 0 | 0.23 | 1.47 | 3.05 | 0.09 |
| CgMYB81 | 0 | 0 | 0 | 0.09 | 0.22 | 0.07 | 0.21 | 0.73 |
| CgMYB82 | 0.26 | 0.12 | 0 | 0.03 | 1.67 | 1.61 | 0.47 | 0.39 |
| CgMYB83 | 1.98 | 1.27 | 6.18 | 3.21 | 0 | 0 | 0 | 0.04 |
| CgMYB84 | 1.43 | 0 | 0 | 0 | 0 | 0 | 0.22 | 0.45 |
| CgMYB85 | 0 | 0 | 0 | 0 | 0 | 0 | 0 | 0 |
| CgMYB86 | 0 | 0 | 0 | 0.03 | 0.11 | 0 | 0.02 | 0 |
| CgMYB87 | 101.84 | 437.59 | 185.65 | 341.76 | 872.47 | 550.24 | 392.75 | 187.66 |
| CgMYB88 | 0 | 0 | 0.09 | 0 | 0 | 0 | 0 | 0 |
| CgMYB89 | 0 | 0 | 0.12 | 0 | 0 | 0 | 0 | 0.06 |
| CgMYB90 | 0.25 | 0.13 | 0.65 | 0.23 | 0.13 | 0.11 | 0.13 | 0.05 |
| CgMYB91 | 1.56 | 7.31 | 81.22 | 6.85 | 0 | 0.08 | 0.04 | 0.05 |
| CgMYB92 | 725.93 | 1005.61 | 830.39 | 623.93 | 431.86 | 364.9 | 360.7 | 270.61 |
| CgMYB93 | 0 | 0 | 0 | 0 | 0 | 0 | 0 | 0.27 |
| CgMYB94 | 33.33 | 21.24 | 7.84 | 12.81 | 0.05 | 0.44 | 1.14 | 28.55 |
| CgMYB95 | 0 | 0 | 0 | 0 | 0.05 | 0.16 | 0.76 | 0.14 |
| CgMYB96 | 0.13 | 0.08 | 0.08 | 0.21 | 1.28 | 1.17 | 0.53 | 0.04 |
| CgMYB97 | 0 | 0.1 | 0.08 | 0.11 | 0.18 | 0.08 | 0.26 | 0.11 |
| CgMYB98 | 0 | 0 | 0 | 0 | 0 | 0.26 | 0 | 0 |
| CgMYB99 | 0 | 0 | 0 | 0 | 0 | 0.26 | 0 | 0 |
| CgMYB100 | 0 | 0 | 0 | 0 | 0 | 0 | 0 | 0 |
| CgMYB101 | 0 | 0.13 | 0 | 0.1 | 0.23 | 0.05 | 0.03 | 0.11 |
| CgMYB102 | 0 | 0 | 0 | 0 | 1.44 | 1.01 | 1.01 | 0.17 |
| CgMYB103 | 0.11 | 0 | 0.37 | 0.64 | 0.15 | 0 | 0 | 0.38 |
| CgMYB104 | 0.64 | 1.67 | 2.25 | 1.01 | 0.61 | 0.81 | 0.81 | 1.09 |

Y-se, sepals of pale-yellow flowers with purple-red spots; Y-pe, petals of pale-yellow flowers with purple-red spots; Y-lip, lips of pale-yellow flowers with purple-red spots; Y-col, columns of pale-yellow flowers with purple-red spots; R-se, sepals of the light purple-red flower; R-pe, petals of the light purple-red flower; R-lip, lips of the light purple-red flower; R-col, columns of the light purple-red flower.

**Supplementary Table 8|** GO annotation information of *CgMYB* proteins.

| **Gene IDs** | **GO IDs** |
| --- | --- |
| CgMYB1 | GO:0003676;GO:0003677;GO:0005488;GO:0005622;GO:0005623;GO:0005634;GO:0043226;GO:0043227;GO:0043229;GO:0043231;GO:0044424;GO:0044464;GO:0097159;GO:1901363 |
| CgMYB2 | GO:0003692;GO:0003677;GO:0005488;GO:0005622;GO:0005623;GO:0005634;GO:0043226;GO:0043227;GO:0043229;GO:0043231;GO:0044424;GO:0044464;GO:0097159;GO:1901363 |
| CgMYB3 | GO:0003703;GO:0003677;GO:0005488;GO:0005622;GO:0005623;GO:0005634;GO:0043226;GO:0043227;GO:0043229;GO:0043231;GO:0044424;GO:0044464;GO:0097159;GO:1901363 |
| CgMYB4 | GO:0003682;GO:0003677;GO:0005488;GO:0005622;GO:0005623;GO:0005634;GO:0043226;GO:0043227;GO:0043229;GO:0043231;GO:0044424;GO:0044464;GO:0097159;GO:1901363 |
| CgMYB5 | ;GO:0003682;GO:0003677;GO:0005488;GO:0005622;GO:0005623;GO:0005634;GO:0043226;GO:0043227;GO:0043229;GO:0043231;GO:0044424;GO:0044464;GO:0097159;GO:1901363 |
| CgMYB6 | GO:0003684;GO:0003677;GO:0005488;GO:0005622;GO:0005623;GO:0005634;GO:0043226;GO:0043227;GO:0043229;GO:0043231;GO:0044424;GO:0044464;GO:0097159;GO:1901363 |
| CgMYB7 | GO:0003679;GO:0003677;GO:0005488;GO:0005622;GO:0005623;GO:0005634;GO:0043226;GO:0043227;GO:0043229;GO:0043231;GO:0044424;GO:0044464;GO:0097159;GO:1901363 |
| CgMYB8 | GO:0003676;GO:0003677;GO:0005488;GO:0005622;GO:0005623;GO:0005634;GO:0043226;GO:0043227;GO:0043229;GO:0043231;GO:0044424;GO:0044464;GO:0097159;GO:1901363 |
| CgMYB9 | GO:0003687;GO:0003677;GO:0005488;GO:0005622;GO:0005623;GO:0005634;GO:0043226;GO:0043227;GO:0043229;GO:0043231;GO:0044424;GO:0044464;GO:0097159;GO:1901363 |
| CgMYB10 | GO:0003677;GO:0003677;GO:0005488;GO:0005622;GO:0005623;GO:0005634;GO:0043226;GO:0043227;GO:0043229;GO:0043231;GO:0044424;GO:0044464;GO:0097159;GO:1901363 |
| CgMYB11 | GO:0003683;GO:0003677;GO:0005488;GO:0005622;GO:0005623;GO:0005634;GO:0043226;GO:0043227;GO:0043229;GO:0043231;GO:0044424;GO:0044464;GO:0097159;GO:1901363 |
| CgMYB12 | GO:0003684;GO:0003677;GO:0005488;GO:0005622;GO:0005623;GO:0005634;GO:0043226;GO:0043227;GO:0043229;GO:0043231;GO:0044424;GO:0044464;GO:0097159;GO:1901363 |
| CgMYB13 | GO:0003685;GO:0003677;GO:0005488;GO:0005622;GO:0005623;GO:0005634;GO:0043226;GO:0043227;GO:0043229;GO:0043231;GO:0044424;GO:0044464;GO:0097159;GO:1901363 |
| CgMYB14 | GO:0003686;GO:0003677;GO:0005488;GO:0005622;GO:0005623;GO:0005634;GO:0043226;GO:0043227;GO:0043229;GO:0043231;GO:0044424;GO:0044464;GO:0097159;GO:1901363 |
| CgMYB15 | GO:0003687;GO:0003677;GO:0005488;GO:0005622;GO:0005623;GO:0005634;GO:0043226;GO:0043227;GO:0043229;GO:0043231;GO:0044424;GO:0044464;GO:0097159;GO:1901363 |
| CgMYB16 | GO:0003688;GO:0003677;GO:0005488;GO:0005622;GO:0005623;GO:0005634;GO:0043226;GO:0043227;GO:0043229;GO:0043231;GO:0044424;GO:0044464;GO:0097159;GO:1901363 |
| CgMYB17 | GO:0003689;GO:0003677;GO:0005488;GO:0005622;GO:0005623;GO:0005634;GO:0043226;GO:0043227;GO:0043229;GO:0043231;GO:0044424;GO:0044464;GO:0097159;GO:1901363 |
| CgMYB18 | GO:0003690;GO:0003677;GO:0003824;GO:0005488;GO:0005622;GO:0005623;GO:0005634;GO:0008168;GO:0016740;GO:0016741;GO:0043226;GO:0043227;GO:0043229;GO:0043231;GO:0044424;GO:0044464;GO:0097159;GO:1901363 |
| CgMYB19 | GO:0003691;GO:0003677;GO:0005488;GO:0005622;GO:0005623;GO:0005634;GO:0043226;GO:0043227;GO:0043229;GO:0043231;GO:0044424;GO:0044464;GO:0097159;GO:1901363 |
| CgMYB20 | GO:0003693;GO:0003677;GO:0005488;GO:0005622;GO:0005623;GO:0005634;GO:0043226;GO:0043227;GO:0043229;GO:0043231;GO:0044424;GO:0044464;GO:0097159;GO:1901363 |
| CgMYB21 | GO:0003694;GO:0003677;GO:0005488;GO:0005622;GO:0005623;GO:0005634;GO:0043226;GO:0043227;GO:0043229;GO:0043231;GO:0044424;GO:0044464;GO:0097159;GO:1901363 |
| CgMYB22 | GO:0003695;GO:0003677;GO:0005488;GO:0005622;GO:0005623;GO:0005634;GO:0043226;GO:0043227;GO:0043229;GO:0043231;GO:0044424;GO:0044464;GO:0097159;GO:1901363 |
| CgMYB23 | GO:0003696;GO:0003677;GO:0005488;GO:0005622;GO:0005623;GO:0005634;GO:0043226;GO:0043227;GO:0043229;GO:0043231;GO:0044424;GO:0044464;GO:0097159;GO:1901363 |
| CgMYB24 | GO:0003697;GO:0003677;GO:0005488;GO:0005622;GO:0005623;GO:0005634;GO:0043226;GO:0043227;GO:0043229;GO:0043231;GO:0044424;GO:0044464;GO:0097159;GO:1901363 |
| CgMYB25 | GO:0003698;GO:0003677;GO:0005488;GO:0005622;GO:0005623;GO:0005634;GO:0043226;GO:0043227;GO:0043229;GO:0043231;GO:0044424;GO:0044464;GO:0097159;GO:1901363 |
| CgMYB26 | GO:0003699;GO:0003677;GO:0005488;GO:0005622;GO:0005623;GO:0005634;GO:0043226;GO:0043227;GO:0043229;GO:0043231;GO:0044424;GO:0044464;GO:0097159;GO:1901363 |
| CgMYB27 | GO:0003700;GO:0003677;GO:0005488;GO:0005622;GO:0005623;GO:0005634;GO:0043226;GO:0043227;GO:0043229;GO:0043231;GO:0044424;GO:0044464;GO:0097159;GO:1901363 |
| CgMYB28 | GO:0003701;GO:0003677;GO:0005488;GO:0005622;GO:0005623;GO:0005634;GO:0043226;GO:0043227;GO:0043229;GO:0043231;GO:0044424;GO:0044464;GO:0097159;GO:1901363 |
| CgMYB29 | GO:0003702;GO:0003677;GO:0005488;GO:0005622;GO:0005623;GO:0005634;GO:0043226;GO:0043227;GO:0043229;GO:0043231;GO:0044424;GO:0044464;GO:0097159;GO:1901363 |
| CgMYB30 | GO:0003704;GO:0003677;GO:0005488;GO:0005622;GO:0005623;GO:0005634;GO:0043226;GO:0043227;GO:0043229;GO:0043231;GO:0044424;GO:0044464;GO:0097159;GO:1901363 |
| CgMYB31 | GO:0003705;GO:0003677;GO:0005488;GO:0005622;GO:0005623;GO:0005634;GO:0043226;GO:0043227;GO:0043229;GO:0043231;GO:0044424;GO:0044464;GO:0097159;GO:1901363 |
| CgMYB32 | GO:0003706;GO:0003677;GO:0005488;GO:0005622;GO:0005623;GO:0005634;GO:0043226;GO:0043227;GO:0043229;GO:0043231;GO:0044424;GO:0044464;GO:0097159;GO:1901363 |
| CgMYB33 | GO:0001067;GO:0003676;GO:0003677;GO:0005488;GO:0005622;GO:0005623;GO:0005634;GO:0009987;GO:0030154;GO:0032502;GO:0043226;GO:0043227;GO:0043229;GO:0043231;GO:0043565;GO:0043565;GO:0044212;GO:0044424;GO:0044464;GO:0048869;GO:0097159;GO:1901363 |
| CgMYB34 | GO:0003676;GO:0003677;GO:0005488;GO:0005622;GO:0005623;GO:0005634;GO:0043226;GO:0043227;GO:0043229;GO:0043231;GO:0044424;GO:0044464;GO:0097159;GO:1901363 |
| CgMYB35 | GO:0003677;GO:0003677;GO:0005488;GO:0005622;GO:0005623;GO:0005634;GO:0016020;GO:0016021;GO:0031224;GO:0043226;GO:0043227;GO:0043229;GO:0043231;GO:0044424;GO:0044425;GO:0044464;GO:0097159;GO:1901363 |
| CgMYB36 | GO:0003678;GO:0003677;GO:0005488;GO:0005622;GO:0005623;GO:0005634;GO:0043226;GO:0043227;GO:0043229;GO:0043231;GO:0044424;GO:0044464;GO:0097159;GO:1901363 |
| CgMYB37 | GO:0003679;GO:0003677;GO:0005488;GO:0005622;GO:0005623;GO:0005634;GO:0043226;GO:0043227;GO:0043229;GO:0043231;GO:0044424;GO:0044464;GO:0097159;GO:1901363 |
| CgMYB38 | GO:0003680;GO:0003677;GO:0005488;GO:0005622;GO:0005623;GO:0005634;GO:0043226;GO:0043227;GO:0043229;GO:0043231;GO:0044424;GO:0044464;GO:0097159;GO:1901363 |
| CgMYB39 | GO:0003681;GO:0003677;GO:0005488;GO:0005622;GO:0005623;GO:0005634;GO:0043226;GO:0043227;GO:0043229;GO:0043231;GO:0044424;GO:0044464;GO:0097159;GO:1901363 |
| CgMYB40 | GO:0003683;GO:0003677;GO:0005488;GO:0005622;GO:0005623;GO:0005634;GO:0043226;GO:0043227;GO:0043229;GO:0043231;GO:0044424;GO:0044464;GO:0097159;GO:1901363 |
| CgMYB41 | GO:0003684;GO:0003677;GO:0005488;GO:0005622;GO:0005623;GO:0005634;GO:0043226;GO:0043227;GO:0043229;GO:0043231;GO:0044424;GO:0044464;GO:0097159;GO:1901363 |
| CgMYB42 | GO:0003685;GO:0003677;GO:0005488;GO:0005622;GO:0005623;GO:0005634;GO:0043226;GO:0043227;GO:0043229;GO:0043231;GO:0044424;GO:0044464;GO:0097159;GO:1901363 |
| CgMYB43 | GO:0001067;GO:0003676;GO:0003677;GO:0005488;GO:0005622;GO:0005623;GO:0005634;GO:0009987;GO:0030154;GO:0032502;GO:0043226;GO:0043227;GO:0043229;GO:0043231;GO:0043565;GO:0043565;GO:0044212;GO:0044424;GO:0044464;GO:0048869;GO:0097159;GO:1901363 |
| CgMYB44 | ;GO:0003676;GO:0003677;GO:0005488;GO:0005622;GO:0005623;GO:0005634;GO:0043226;GO:0043227;GO:0043229;GO:0043231;GO:0044424;GO:0044464;GO:0097159;GO:1901363 |
| CgMYB45 | ;GO:0003677;GO:0003677;GO:0005488;GO:0005622;GO:0005623;GO:0005634;GO:0043226;GO:0043227;GO:0043229;GO:0043231;GO:0044424;GO:0044464;GO:0097159;GO:1901363 |
| CgMYB46 | ;GO:0003678;GO:0003677;GO:0005488;GO:0005622;GO:0005623;GO:0005634;GO:0043226;GO:0043227;GO:0043229;GO:0043231;GO:0044424;GO:0044464;GO:0097159;GO:1901363 |
| CgMYB47 | ;GO:0003679;GO:0003677;GO:0005488;GO:0005622;GO:0005623;GO:0005634;GO:0043226;GO:0043227;GO:0043229;GO:0043231;GO:0044424;GO:0044464;GO:0097159;GO:1901363 |
| CgMYB48 | ;GO:0003680;GO:0003677;GO:0005488;GO:0005622;GO:0005623;GO:0005634;GO:0043226;GO:0043227;GO:0043229;GO:0043231;GO:0044424;GO:0044464;GO:0097159;GO:1901363 |
| CgMYB49 | ;GO:0003681;GO:0003677;GO:0005488;GO:0005622;GO:0005623;GO:0005634;GO:0043226;GO:0043227;GO:0043229;GO:0043231;GO:0044424;GO:0044464;GO:0097159;GO:1901363 |
| CgMYB50 | ;GO:0003683;GO:0003677;GO:0005488;GO:0005622;GO:0005623;GO:0005634;GO:0043226;GO:0043227;GO:0043229;GO:0043231;GO:0044424;GO:0044464;GO:0097159;GO:1901363 |
| CgMYB51 | GO:0000272;GO:0003676;GO:0003677;GO:0003824;GO:0004553;GO:0005488;GO:0005622;GO:0005623;GO:0005634;GO:0005975;GO:0005975;GO:0005975;GO:0006520;GO:0006807;GO:0008144;GO:0008152;GO:0009056;GO:0009057;GO:0009987;GO:0016052;GO:0016160;GO:0016161;GO:0016740;GO:0016741;GO:0016740;GO:0016741;GO:0016740;GO:0019752;GO:0019842;GO:0030170;GO:0036094;GO:0043167;GO:0043168;GO:0043170;GO:0043226;GO:0043227;GO:0043229;GO:0043231;GO:0043436;GO:0044237;GO:0044238;GO:0044281;GO:0044424;GO:0044464;GO:0048037;GO:0050662;GO:0070279;GO:0071704;GO:0097159;GO:0102229;GO:1901363;GO:1901564;GO:1901575 |
| CgMYB52 | GO:0003676;GO:0003677;GO:0005488;GO:0005622;GO:0005623;GO:0005634;GO:0043226;GO:0043227;GO:0043229;GO:0043231;GO:0044424;GO:0044464;GO:0097159;GO:1901363 |
| CgMYB53 | GO:0003677;GO:0003677;GO:0005488;GO:0005622;GO:0005623;GO:0005634;GO:0043226;GO:0043227;GO:0043229;GO:0043231;GO:0044424;GO:0044464;GO:0097159;GO:1901363 |
| CgMYB54 | GO:0003678;GO:0003677;GO:0005488;GO:0005622;GO:0005623;GO:0005634;GO:0043226;GO:0043227;GO:0043229;GO:0043231;GO:0044424;GO:0044464;GO:0097159;GO:1901363 |
| CgMYB55 | GO:0003679;GO:0003677;GO:0005488;GO:0005622;GO:0005623;GO:0005634;GO:0043226;GO:0043227;GO:0043229;GO:0043231;GO:0044424;GO:0044464;GO:0097159;GO:1901363 |
| CgMYB56 | GO:0003680;GO:0003677;GO:0005488;GO:0005622;GO:0005623;GO:0005634;GO:0043226;GO:0043227;GO:0043229;GO:0043231;GO:0044424;GO:0044464;GO:0097159;GO:1901363 |
| CgMYB57 | GO:0003681;GO:0003677;GO:0005488;GO:0005622;GO:0005623;GO:0005634;GO:0043226;GO:0043227;GO:0043229;GO:0043231;GO:0044424;GO:0044464;GO:0097159;GO:1901363 |
| CgMYB58 | GO:0003682;GO:0003677;GO:0005488;GO:0005622;GO:0005623;GO:0005634;GO:0043226;GO:0043227;GO:0043229;GO:0043231;GO:0044424;GO:0044464;GO:0097159;GO:1901363 |
| CgMYB59 | GO:0003683;GO:0003677;GO:0005488;GO:0005622;GO:0005623;GO:0005634;GO:0043226;GO:0043227;GO:0043229;GO:0043231;GO:0044424;GO:0044464;GO:0097159;GO:1901363 |
| CgMYB60 | GO:0003685;GO:0003677;GO:0005488;GO:0005622;GO:0005623;GO:0005634;GO:0043226;GO:0043227;GO:0043229;GO:0043231;GO:0044424;GO:0044464;GO:0097159;GO:1901363 |
| CgMYB61 | GO:0003686;GO:0003677;GO:0005488;GO:0005622;GO:0005623;GO:0005634;GO:0043226;GO:0043227;GO:0043229;GO:0043231;GO:0044424;GO:0044464;GO:0097159;GO:1901363 |
| CgMYB62 | GO:0003687;GO:0003677;GO:0005488;GO:0005622;GO:0005623;GO:0005634;GO:0043226;GO:0043227;GO:0043229;GO:0043231;GO:0044424;GO:0044464;GO:0097159;GO:1901363 |
| CgMYB63 | GO:0003688;GO:0003677;GO:0005488;GO:0005622;GO:0005623;GO:0005634;GO:0043226;GO:0043227;GO:0043229;GO:0043231;GO:0044424;GO:0044464;GO:0097159;GO:1901363 |
| CgMYB64 | GO:0003689;GO:0003677;GO:0005488;GO:0005622;GO:0005623;GO:0005634;GO:0043226;GO:0043227;GO:0043229;GO:0043231;GO:0044424;GO:0044464;GO:0097159;GO:1901363 |
| CgMYB65 | GO:0003690;GO:0003677;GO:0005488;GO:0005622;GO:0005623;GO:0005634;GO:0043226;GO:0043227;GO:0043229;GO:0043231;GO:0044424;GO:0044464;GO:0097159;GO:1901363 |
| CgMYB66 | GO:0000272;GO:0003676;GO:0003677;GO:0003824;GO:0004553;GO:0005488;GO:0005622;GO:0005623;GO:0005634;GO:0005975;GO:0005975;GO:0005975;GO:0006520;GO:0006807;GO:0008144;GO:0008152;GO:0009056;GO:0009057;GO:0009987;GO:0016052;GO:0016160;GO:0016161;GO:0016740;GO:0016741;GO:0016740;GO:0016741;GO:0016740;GO:0019752;GO:0019842;GO:0030170;GO:0036094;GO:0043167;GO:0043168;GO:0043170;GO:0043226;GO:0043227;GO:0043229;GO:0043231;GO:0043436;GO:0044237;GO:0044238;GO:0044281;GO:0044424;GO:0044464;GO:0048037;GO:0050662;GO:0070279;GO:0071704;GO:0097159;GO:0102229;GO:1901363;GO:1901564;GO:1901575 |
| CgMYB67 | GO:0003676;GO:0003677;GO:0005488;GO:0005622;GO:0005623;GO:0005634;GO:0043226;GO:0043227;GO:0043229;GO:0043231;GO:0044424;GO:0044464;GO:0097159;GO:1901363 |
| CgMYB68 | GO:0003677;GO:0003677;GO:0005488;GO:0005622;GO:0005623;GO:0005634;GO:0043226;GO:0043227;GO:0043229;GO:0043231;GO:0044424;GO:0044464;GO:0097159;GO:1901363 |
| CgMYB69 | GO:0003678;GO:0003677;GO:0005488;GO:0005622;GO:0005623;GO:0005634;GO:0043226;GO:0043227;GO:0043229;GO:0043231;GO:0044424;GO:0044464;GO:0097159;GO:1901363 |
| CgMYB70 | GO:0003680;GO:0003677;GO:0005488;GO:0005622;GO:0005623;GO:0005634;GO:0043226;GO:0043227;GO:0043229;GO:0043231;GO:0044424;GO:0044464;GO:0097159;GO:1901363 |
| CgMYB71 | GO:0003681;GO:0003677;GO:0005488;GO:0005622;GO:0005623;GO:0005634;GO:0043226;GO:0043227;GO:0043229;GO:0043231;GO:0044424;GO:0044464;GO:0097159;GO:1901363 |
| CgMYB72 | GO:0003682;GO:0003677;GO:0005488;GO:0005622;GO:0005623;GO:0005634;GO:0043226;GO:0043227;GO:0043229;GO:0043231;GO:0044424;GO:0044464;GO:0097159;GO:1901363 |
| CgMYB73 | GO:0003683;GO:0003677;GO:0005488;GO:0005622;GO:0005623;GO:0005634;GO:0043226;GO:0043227;GO:0043229;GO:0043231;GO:0044424;GO:0044464;GO:0097159;GO:1901363 |
| CgMYB74 | GO:0003684;GO:0003677;GO:0005488;GO:0005622;GO:0005623;GO:0005634;GO:0043226;GO:0043227;GO:0043229;GO:0043231;GO:0044424;GO:0044464;GO:0097159;GO:1901363 |
| CgMYB75 | GO:0000003;GO:0001101;GO:0001558;GO:0003676;GO:0003006;GO:0003677;GO:0003700;GO:0005488;GO:0005622;GO:0005623;GO:0005634;GO:0006355;GO:0006996;GO:0007033;GO:0007275;GO:0007276;GO:0009555;GO:0009719;GO:0009725;GO:0009739;GO:0009787;GO:0009889;GO:0009891;GO:0009893;GO:0009966;GO:0009967;GO:0009987;GO:0010033;GO:0010468;GO:0010556;GO:0010557;GO:0010604;GO:0010628;GO:0010646;GO:0010647;GO:0010941;GO:0010942;GO:0016043;GO:0016741;GO:0019222;GO:0019953;GO:0022412;GO:0022603;GO:0022604;GO:0022414;GO:0023051;GO:0023056;GO:0030154;GO:0031323;GO:0031325;GO:0031326;GO:0031328;GO:0032501;GO:0032502;GO:0032504;GO:0033993;GO:0040008;GO:0042221;GO:0042995;GO:0043067;GO:0043068;GO:0043226;GO:0043227;GO:0043229;GO:0043231;GO:0043900;GO:0044424;GO:0044464;GO:0044703;GO:0045893;GO:0045935;GO:0048229;GO:0048232;GO:0048235;GO:0048522;GO:0048518;GO:0048584;GO:0048583;GO:0048609;GO:0048831;GO:00486381;GO:0048869;GO:0050789;GO:0050793;GO:0050794;GO:0050896;GO:0051128;GO:0051171;GO:0051173;GO:0051239;GO:0051252;GO:0051254;GO:0051510;GO:0051704;GO:0055046;GO:0060255;GO:0065007;GO:0071840;GO:0080090;GO:0090406;GO:0097159;GO:0120025;GO:0140110;GO:1901363;GO:1901419;GO:1901700;GO:1902680;GO:1903508;GO:1903506;GO:1905957;GO:2000024;GO:2000026;GO:2000112;GO:2000241;GO:2001141 |
| CgMYB76 | GO:0003676;GO:0003677;GO:0005488;GO:0005622;GO:0005623;GO:0005634;GO:0043226;GO:0043227;GO:0043229;GO:0043231;GO:0044424;GO:0044464;GO:0097159;GO:1901363 |
| CgMYB77 | GO:0003677;GO:0003677;GO:0005488;GO:0005622;GO:0005623;GO:0005634;GO:0043226;GO:0043227;GO:0043229;GO:0043231;GO:0044424;GO:0044464;GO:0097159;GO:1901363 |
| CgMYB78 | GO:0003678;GO:0003677;GO:0005488;GO:0005622;GO:0005623;GO:0005634;GO:0043226;GO:0043227;GO:0043229;GO:0043231;GO:0044424;GO:0044464;GO:0097159;GO:1901363 |
| CgMYB79 | GO:0000272;GO:0003676;GO:0003677;GO:0003824;GO:0004553;GO:0005488;GO:0005622;GO:0005623;GO:0005634;GO:0005975;GO:0005975;GO:0005975;GO:0006520;GO:0006807;GO:0008144;GO:0008152;GO:0009056;GO:0009057;GO:0009987;GO:0016052;GO:0016160;GO:0016161;GO:0016740;GO:0016741;GO:0016740;GO:0016741;GO:00167401;GO:0019842;GO:0030170;GO:0036094;GO:0043167;GO:0043168;GO:0043170;GO:0043226;GO:0043227;GO:0043229;GO:0043231;GO:0043436;GO:0044237;GO:0044238;GO:0044281;GO:0044424;GO:0044464;GO:0048037;GO:0050662;GO:0070279;GO:0071704;GO:0097159;GO:0102229;GO:1901363;GO:1901564;GO:1901575 |
| CgMYB80 | GO:0003677;GO:0003677;GO:0005488;GO:0005622;GO:0005623;GO:0005634;GO:0043226;GO:0043227;GO:0043229;GO:0043231;GO:0044424;GO:0044464;GO:0097159;GO:1901363 |
| CgMYB81 | GO:0003678;GO:0003677;GO:0005488;GO:0005622;GO:0005623;GO:0005634;GO:0043226;GO:0043227;GO:0043229;GO:0043231;GO:0044424;GO:0044464;GO:0097159;GO:1901363 |
| CgMYB82 | GO:0003679;GO:0003677;GO:0005488;GO:0005622;GO:0005623;GO:0005634;GO:0043226;GO:0043227;GO:0043229;GO:0043231;GO:0044424;GO:0044464;GO:0097159;GO:1901363 |
| CgMYB83 | GO:0003680;GO:0003677;GO:0005488;GO:0005622;GO:0005623;GO:0005634;GO:0043226;GO:0043227;GO:0043229;GO:0043231;GO:0044424;GO:0044464;GO:0097159;GO:1901363 |
| CgMYB84 | GO:0003681;GO:0003677;GO:0005488;GO:0005622;GO:0005623;GO:0005634;GO:0043226;GO:0043227;GO:0043229;GO:0043231;GO:0044424;GO:0044464;GO:0097159;GO:1901363 |
| CgMYB85 | GO:0003682;GO:0003677;GO:0005488;GO:0005622;GO:0005623;GO:0005634;GO:0043226;GO:0043227;GO:0043229;GO:0043231;GO:0044424;GO:0044464;GO:0097159;GO:1901363 |
| CgMYB86 | GO:0003683;GO:0003677;GO:0005488;GO:0005622;GO:0005623;GO:0005634;GO:0043226;GO:0043227;GO:0043229;GO:0043231;GO:0044424;GO:0044464;GO:0097159;GO:1901363 |
| CgMYB87 | GO:0003684;GO:0003677;GO:0005488;GO:0005622;GO:0005623;GO:0005634;GO:0043226;GO:0043227;GO:0043229;GO:0043231;GO:0044424;GO:0044464;GO:0097159;GO:1901363 |
| CgMYB88 | GO:0003685;GO:0003677;GO:0005488;GO:0005622;GO:0005623;GO:0005634;GO:0043226;GO:0043227;GO:0043229;GO:0043231;GO:0044424;GO:0044464;GO:0097159;GO:1901363 |
| CgMYB89 | GO:0003686;GO:0003677;GO:0005488;GO:0005622;GO:0005623;GO:0005634;GO:0043226;GO:0043227;GO:0043229;GO:0043231;GO:0044424;GO:0044464;GO:0097159;GO:1901363 |
| CgMYB91 | GO:0003688;GO:0003677;GO:0005488;GO:0005622;GO:0005623;GO:0005634;GO:0043226;GO:0043227;GO:0043229;GO:0043231;GO:0044424;GO:0044464;GO:0097159;GO:1901363 |
| CgMYB92 | GO:0003689;GO:0003677;GO:0005488;GO:0005622;GO:0005623;GO:0005634;GO:0043226;GO:0043227;GO:0043229;GO:0043231;GO:0044424;GO:0044464;GO:0097159;GO:1901363 |
| CgMYB93 | GO:0003690;GO:0003677;GO:0005488;GO:0005622;GO:0005623;GO:0005634;GO:0043226;GO:0043227;GO:0043229;GO:0043231;GO:0044424;GO:0044464;GO:0097159;GO:1901363 |
| CgMYB94 | GO:0003691;GO:0003677;GO:0005488;GO:0005622;GO:0005623;GO:0005634;GO:0043226;GO:0043227;GO:0043229;GO:0043231;GO:0044424;GO:0044464;GO:0097159;GO:1901363 |
| CgMYB95 | GO:0003692;GO:0003677;GO:0005488;GO:0005622;GO:0005623;GO:0005634;GO:0043226;GO:0043227;GO:0043229;GO:0043231;GO:0044424;GO:0044464;GO:0097159;GO:1901363 |
| CgMYB96 | GO:0003693;GO:0003677;GO:0005488;GO:0005622;GO:0005623;GO:0005634;GO:0043226;GO:0043227;GO:0043229;GO:0043231;GO:0044424;GO:0044464;GO:0097159;GO:1901363 |
| CgMYB97 | GO:0003694;GO:0003677;GO:0005488;GO:0005622;GO:0005623;GO:0005634;GO:0043226;GO:0043227;GO:0043229;GO:0043231;GO:0044424;GO:0044464;GO:0097159;GO:1901363 |
| CgMYB98 | GO:0003695;GO:0003677;GO:0005488;GO:0005622;GO:0005623;GO:0005634;GO:0043226;GO:0043227;GO:0043229;GO:0043231;GO:0044424;GO:0044464;GO:0097159;GO:1901363 |
| CgMYB99 | GO:0003696;GO:0003677;GO:0005488;GO:0005622;GO:0005623;GO:0005634;GO:0043226;GO:0043227;GO:0043229;GO:0043231;GO:0044424;GO:0044464;GO:0097159;GO:1901363 |
| CgMYB100 | GO:0003678;GO:0003677;GO:0005488;GO:0005622;GO:0005623;GO:0005634;GO:0043226;GO:0043227;GO:0043229;GO:0043231;GO:0044424;GO:0044464;GO:0097159;GO:1901363 |
| CgMYB101 | GO:0003679;GO:0003677;GO:0005488;GO:0005622;GO:0005623;GO:0005634;GO:0016020;GO:0016021;GO:0031224;GO:0043226;GO:0043227;GO:0043229;GO:0043231;GO:0044424;GO:0044425;GO:0044464;GO:0097159;GO:1901363 |
| CgMYB102 | GO:0003680;GO:0003677;GO:0005488;GO:0005622;GO:0005623;GO:0005634;GO:0043226;GO:0043227;GO:0043229;GO:0043231;GO:0044424;GO:0044464;GO:0097159;GO:1901363 |
| CgMYB103 | GO:0003681;GO:0003677;GO:0005488;GO:0005622;GO:0005623;GO:0005634;GO:0043226;GO:0043227;GO:0043229;GO:0043231;GO:0044424;GO:0044464;GO:0097159;GO:1901363 |
| CgMYB104 | GO:0003682;GO:0003677;GO:0005488;GO:0005622;GO:0005623;GO:0005634;GO:0043226;GO:0043227;GO:0043229;GO:0043231;GO:0044424;GO:0044464;GO:0097159;GO:1901363 |

**Supplementary Table 9|** The expression values of *CgMYB* genes in different flower tissues.

| **gene** | **Y-se** | **Y-pe** | **Y-lip** | **Y-col** | **R-se** | **R-pe** | **R-lip** | **R-col** |
| --- | --- | --- | --- | --- | --- | --- | --- | --- |
| CgMYB32 | 23.688 | 24.395 | 22.405 | 21.922 | 21.795 | 21.455 | 23.774 | 24.371 |
|  | 23.606 | 23.637 | 22.197 | 21.853 | 21.581 | 21.407 | 23.780 | 24.637 |
|  | 23.512 | 23.611 | 22.247 | 21.723 | 21.604 | 21.200 | 23.559 | 24.221 |
| CgMYB91 | 34.644 | 33.116 | 28.920 | 34.028 | 36.169 | 34.552 | 33.104 | 36.426 |
|  | 34.669 | 32.867 | 28.952 | 33.728 | 35.586 | 34.767 | 33.124 | 36.080 |
|  | 34.652 | 32.998 | 29.1417 | 33.822 | 36.296 | 34.023 | 33.175 | 35.682 |

The values in the table are the mean CT values of three technical replicates for different floral tissues, with three biological replicates used for each flower tissue.

Y-se, sepals of pale-yellow flowers with purple-red spots; Y-pe, petals of pale-yellow flowers with purple-red spots; Y-lip, lips of pale-yellow flowers with purple-red spots; Y-col, columns of pale-yellow flowers with purple-red spots; R-se, sepals of the light purple-red flower; R-pe, petals of the light purple-red flower; R-lip, lips of the light purple-red flower; R-col, columns of the light purple-red flower.
